# Soybean Oil Is More Obesogenic and Diabetogenic than Coconut Oil and Fructose in Mouse: Potential Role for the Liver

**DOI:** 10.1371/journal.pone.0132672

**Published:** 2015-07-22

**Authors:** Poonamjot Deol, Jane R. Evans, Joseph Dhahbi, Karthikeyani Chellappa, Diana S. Han, Stephen Spindler, Frances M. Sladek

**Affiliations:** 1 Department of Cell Biology and Neuroscience, University of California, Riverside, Riverside, California, United States of America; 2 Department of Biochemistry, University of California, Riverside, Riverside, California, United States of America; East Tennessee State University, UNITED STATES

## Abstract

The obesity epidemic in the U.S. has led to extensive research into potential contributing dietary factors, especially fat and fructose. Recently, increased consumption of soybean oil, which is rich in polyunsaturated fatty acids (PUFAs), has been proposed to play a causal role in the epidemic. Here, we designed a series of four isocaloric diets (HFD, SO-HFD, F-HFD, F-SO-HFD) to investigate the effects of saturated versus unsaturated fat, as well as fructose, on obesity and diabetes. C57/BL6 male mice fed a diet moderately high in fat from coconut oil and soybean oil (SO-HFD, 40% kcal total fat) showed statistically significant increases in weight gain, adiposity, diabetes, glucose intolerance and insulin resistance compared to mice on a diet consisting primarily of coconut oil (HFD). They also had fatty livers with hepatocyte ballooning and very large lipid droplets as well as shorter colonic crypt length. While the high fructose diet (F-HFD) did not cause as much obesity or diabetes as SO-HFD, it did cause rectal prolapse and a very fatty liver, but no balloon injury. The coconut oil diet (with or without fructose) increased spleen weight while fructose in the presence of soybean oil increased kidney weight. Metabolomics analysis of the liver showed an increased accumulation of PUFAs and their metabolites as well as γ-tocopherol, but a decrease in cholesterol in SO-HFD. Liver transcriptomics analysis revealed a global dysregulation of cytochrome P450 (*Cyp*) genes in SO-HFD versus HFD livers, most notably in the *Cyp3a* and *Cyp2c* families. Other genes involved in obesity (e.g., *Cidec*, *Cd36*), diabetes (*Igfbp1*), inflammation (*Cd63*), mitochondrial function (*Pdk4*) and cancer (*H19*) were also upregulated by the soybean oil diet. Taken together, our results indicate that in mice a diet high in soybean oil is more detrimental to metabolic health than a diet high in fructose or coconut oil.

## Introduction

There has been an alarming increase in obesity and its associated co-morbidities—diabetes and heart disease—in the U.S. during the last four decades. Recent estimates suggest that 36% of the U.S. population is currently obese and by 2030 this will increase to ~50% [1,2]. Furthermore, obesity is no longer a problem of developed countries but has become a major global health issue [3] with an estimated 3.4 million deaths worldwide being attributed to it annually [4]. Often associated with obesity are diabetes, insulin resistance (IR) and nonalcoholic fatty liver disease (NAFLD), which along with heart disease and hypertension, are referred to as the Metabolic Syndrome [5]. There are many contributing factors to obesity, including genetics, lifestyle, environmental factors and microbiota, but diet is still one of the most relevant, both in terms of the number of calories that are consumed as well as the source of those calories.

Saturated fatty acids (SFAs) were deemed unhealthy due to studies in the 1950s and 1960s that showed a positive correlation between dietary SFAs and the risk for cardiovascular disease [6,7]. As a result, nutritional guidelines were developed that encouraged people to reduce their intake of saturated fat, typically found in meat and dairy products, and increase their intake of polyunsaturated fatty acids (PUFAs) found in plant oils [8,9]. These guidelines are still in effect today [10]. In recent years, however, there has been a shift in the dialogue surrounding which dietary fats are the most harmful, with some studies suggesting a reconsideration of nutritional guidelines [11,12]. In particular, there is a growing body of evidence that suggests that saturated fat from sources such as coconut and palm oil, which are rich in medium chain triglycerides (MCTs), may actually be beneficial for the prevention and treatment of the Metabolic Syndrome [13–15].

The recommendation for decreased saturated fat consumption, as well as other factors, led to a dramatic, >1000% increase in the consumption of soybean oil in the U.S. from 0.01 to 11.6 kg/yr/capita between 1909–1999 [16]. Approximately 40 million tons of soybean oil were produced worldwide in 2007, which is about one half of all the edible vegetable oil and one-third of all fats and seed oils produced [17]. Soybean oil is heavily used in processed foods, margarines, salad dressings and snack foods, and is the oil of choice in many restaurants and fast food establishments [18]. While there has been extensive investigation of the role of various other dietary components in obesity, especially SFAs, soybean oil has received relatively little attention. Soybean oil is high in PUFAs whereas most experimental animal studies employ diets high in saturated fats, typically lard. While several studies have looked at the effects of soybean oil on obesity and diabetes, the experimental designs were quite variable as were the results [19–25]. There has also not been any global transcriptomics or metabolomics analysis of the effects of soybean oil on the liver, a primary metabolic organ.

Another component of the American diet that has increased substantially in the last four decades is fructose, primarily in the form of high fructose corn syrup (HFCS) in processed foods and sodas [26–28]. Fructose consumption has increased from ~37 g/day in 1977 [29] to ~49 g/day in 2004, with the highest intake among teenage males (75 g/day) [30]. Despite extensive investigation of the effects of HFCS, especially in human studies (reviewed in [28,31]), there is still a debate over the role of fructose in the development of obesity and the Metabolic Syndrome: while some studies link the increase in obesity to an increased use of HFCS in the American diet [32], others cite a lack of definitive clinical evidence on the topic [33]. Aside from one recent study that examined the effects of soybean oil and fructose on butyrate production in rat intestine [34], we found no reports in the literature that compared the metabolic effects of HFCS to those of soybean oil, nor any that examined these two components together.

In this study, we examined the effect of both soybean oil and fructose on the development of obesity and its associated co-morbidities in C57/BL6 male mice, in the context of a diet moderately high in total fat. We formulated diets with 40% kcal fat from coconut oil (HFD) or coconut oil plus soybean oil (SO-HFD) and then supplemented those with fructose (F-HFD and F-SO-HFD). The total amount of fat is comparable to current American consumption [35] as is the soybean oil [16,36] and fructose [37]. Mice on the high soybean oil diet (SO-HFD) showed increased weight gain, adiposity, fatty liver with balloon injury, diabetes and IR, compared to mice on the HFD. Fructose in the diet (F-HFD and F-SO-HFD) had less severe metabolic effects than soybean oil but caused rectal prolapse and seemed to synergize with soybean oil to increase kidney weight. The SO-HFD produced distinct changes in the liver transcriptome from the HFD, most notable of which was a global dysregulation of cytochrome P450 (*Cyp*) genes as well as genes involved in obesity, diabetes, lipid metabolism and cancer. Metabolomic analyses of the HFD and SO-HFD livers revealed a greater accumulation of PUFAs and their metabolites but also increased antioxidant capacity and decreased cholesterol in SO-HFD. Taken together, these results indicate that while there may be some health benefits to a diet rich in soybean oil, and while fructose induced some negative effects in the gut and kidney, overall soybean oil induced more obesity, diabetes, IR and liver injury than either fructose or saturated fat from coconut oil in mice.

## Materials and Methods

### Animals

Male C57/BL6 mice were weaned at three weeks of age and assigned randomly to one of the five diets used in the study. The animals were maintained on a 12:12 h light-dark cycle in a non-specific pathogen free vivarium at the University of California, Riverside (UCR). Twelve mice were put on each diet with three to four animals per cage. Individual mouse weights were recorded once a week.

### Ethics Statement

Care and treatment of animals was in accordance with guidelines from and approved by the University of California Riverside Institutional Animal Care and Use Committee (AUP#20110015). All mice had *ad libitum* access to food and water (other than the indicated fasting times). At the end of the study, mice were euthanized by carbon dioxide inhalation, in accordance with stated NIH guidelines.

### Diets

Four isocaloric diets with 4.87 kcal/gm (5.56 kcal total) (Table 1) were formulated in conjunction with Research Diets, Inc. (New Brunswick, NJ). The diets are based on the Surwit diet, which is widely used in diet-induced obesity studies and formulated with elements from the AIN-93 diet. The 5% fiber from cellulose in the AIN diet is replaced with cornstarch [38].

**Table 1:** Composition of diets used in this study.

	**Viv**	**HFD**	**SO-HFD**	**F-HFD**	**F-SO-HFD**
**Nutrient**	**gm%**	**gm%**	**gm%**	**gm%**	**gm%**
Protein	23.9	20.1	20.1	20.1	20.1
Carbohydrate	48.7	53.4	53.4	53.4	53.4
Fat	5.0	21.5	21.5	21.5	21.5
*kcal/gm*	*3*.*36*	*4*.*87*	*4*.*87*	*4*.*87*	*4*.*87*
**Ingredient** ^**a**^		**gm**	**gm**	**gm**	**gm**
Casein, 80 Mesh		228	228	228	228
DL-Methionine		2	2	2	2
Maltodextrin 10		120	120	120	120
Corn Starch		480	480	125	125
Fructose		0	0	355	355
Soybean Oil		25	115	25	115
Coconut Oil, Hydrogenated		220	130	220	130
Mineral Mix S10001		40	40	40	40
Sodium Bicarbonate		10.5	10.5	10.5	10.5
Potassium Citrate, 1 H2O		4	4	4	4
Vitamin Mix V10001		40	40	40	40
Choline Bitartrate		2	2	2	2
*Total*		1141.55	1141.55	1141.55	1141.55
**Key Variables**	**kcal%**	**kcal%**	**kcal%**	**kcal%**	**kcal%**
Linoleic Acid	1.2	2.2	10	2.2	10
Fructose	0.3	0.4	0.4	25.9	25.9
Total fat	13.5	40	40	40	40

**^a^**Individual ingredients for the vivarium chow can be found at http://www.newcolab.com/docs/5001.pdf

The basic high fat diet (HFD) had 40 kcal% total fat with 36 kcal% from coconut oil and 4 kcal% from soybean oil. A small amount of soybean oil was added to all HFDs to provide the essential fatty acids linoleic acid (LA, C18:2) and α-linolenic acid (C18:3) (Table 2) [39–41]. In the high soybean oil diet (SO-HFD) a portion of the coconut oil was replaced with soybean oil to give a final concentration of 21 kcal% fat calories from coconut oil and 19 kcal% from soybean oil, of which 10 kcal% were from LA. (Soybean oil is ~55% LA [42]). The high fructose diets (F-HFD and F-SO-HFD) had 25.9 kcal% of energy from added fructose and the same fatty acid composition as HFD and SO-HFD, respectively. The total amounts of carbohydrates and protein were constant across all the diets. Regular vivarium (Viv) chow (Purina Test Diet 5001, Newco Distributors, Rancho Cucamonga, CA) was used as a low fat control. Diets were provided in pellet form, twice weekly for up to 16 or 35 weeks; the amount of food consumed was monitored on a per cage basis.

**Table 2:** Fatty acid profile of oils used in diets in this study.

	**HFD F-HFD**	**SO-HFD F-SO-HFD**
**Source of fat**	**kcal%**	**kcal%**
Coconut Oil	220	130
Soybean Oil	25	115
*Total (grams)*	*245*	*245*
**Specific fatty acids** ^**a**^		
C6, Caproic	1.3	0.8
C8, Caprylic	16.9	10
C10, Capric	13	7.7
C12, Lauric	104.7	61.9
C14, Myristic	39.6	23.4
C16, Palmitic	21.7	23.3
C18, Stearic	24.3	18.2
C18:1, Oleic	7.8	29
C18:2, Linoleic	13.4	61.6
C18:3, Linolenic	2	9
*Total (grams)*	*244*.*8*	*244*.*7*
**Type of fats**		
Saturated (grams)	221.6	145.2
Monounsaturated (grams)	7.8	29
Polyunsaturated (grams)	15.4	70.53
Saturated (%)	90.5	59.3
Monounsaturated (%)	3.2	11.8
Polyunsaturated (%)	6.3	28.83
Linoleic Acid (%)	5.5	25.16
Oleic Acid (%)	3.2	11.8
Alpha-linolenic acid (%)	0.8	3.7
Total Omega-3 (%)	0.8	3.7

^a^ Fatty acids not listed are below the limit of detection

### Glucose and Insulin Tolerance Tests

To measure glucose tolerance (GTT), mice were fasted overnight (~18 h) according to the standard operating procedures outlined by the NIH Mouse Metabolic Phenotyping Center Consortium [43] and glucose (2 g/kg body weight) was administered by intraperitoneal (IP) injection of a 20% glucose solution in 0.9% saline. Tail blood glucose was measured at 0 (pre-injection), 15, 30, 60 and 120 min after injection using OneTouch Ultra Glucose Meter and OneTouch Ultra Test Strips (LifeScan Inc, Milpitas, CA). To measure insulin sensitivity (ITT), mice were fasted for 4.5 h and then injected IP with 0.75 U/kg of Humulin R (Eli Lilly and Company, Indianapolis, IN). Tail blood glucose was measured at 0, 15, 30, 60 and 90 min as for the GTT.

### Tissue Samples and Staining

Tissues were collected and snap frozen in liquid nitrogen before storage at -80°C or fixed in 10% neutral-buffered formalin for 24 h before storing in 30% sucrose solution at 4°C. Liver tissue was put into RNA*later* (Ambion-Life Technologies, Carlsbad, CA) for 24 h before storage at -80°C. Mesenteric, peri-renal, gonadal and flank subcutaneous adipose tissues were excised and weighed. Frozen liver tissues were sectioned at 5 μm on a Microm cryostat (Thermo Scientific, Waltham, MA) set to −19°C and then air-dried. Rehydrated liver sections were placed in 100% propylene glycol for 2 min, and stained in 0.5% Oil Red O (Sigma-Aldrich, St. Louis, MO) solution in propylene glycol for 10 min at 60°C. Slides were transferred to an 85% propylene glycol solution for 2 min and rinsed twice with distilled water. The slides were counterstained with Mayer’s hematoxylin for 40 sec, rinsed in running tap water for 3 min followed by 30 sec in distilled water and mounted with glycerin jelly. Images were captured at 40X (Zeiss Axioplan).

### RNA Extraction and Sequencing

Total RNA was isolated from liver samples using a miRNeasy kit (Qiagen, Inc., Valencia, CA) and evaluated for purity and concentration by NanoDrop (Wilmington, DE) and Agilent 2100 Bioanalyzer (Santa Clara, CA). Poly(A)^+^ RNA (4 μg) with an RNA Integrity Number (RIN) of 7.6 or higher was used to construct sequencing libraries with the TruSeq Long RNA Sample Prep Kit (Illumina, San Diego, CA). Libraries were validated by Bioanalyzer, pooled in equimolar amounts, and sequenced on an Illumina HiSeq 2000 at the UCR Genomics Core to generate 50 base, paired-end reads. Three biological replicates from livers (large lobe) of mice fed Viv, HFD or SO-HFD for 35 weeks were sequenced twice (nine samples per lane) yielding in >70 million reads per sample, more than twice the recommended number for mammalian tissues [44].

### Bioinformatic Analysis

Paired-end sequencing reads were aligned to the mouse reference genome (GRCm38/mm10 assembly) with Tophat v1.2 [45] and processed by Cufflinks [46] to assemble transcripts and measure their relative abundances in FPKM units (fragments per kilobase of exon per million fragments mapped). Assembled transcripts from experimental samples were compared with the RefSeq refFlat annotated transcriptome downloaded from the UCSC Genome Browser and examined for differential expression using the Cuffcompare and Cuffdiff utilities included in the Cufflinks package. Cuffdiff was run with FPKM upper-quartile normalization and a false discovery rate (FDR, *q*-value) threshold of 5%. Dysregulated genes (≥ 1.5 log2 fold change) in SO-HFD versus HFD livers were uploaded to DAVID (http://david.abcc.ncifcrf.gov) for functional annotation clustering [47]. Lists of various disease-associated mouse genes were generated using Pubmed Genes (searched for obesity, diabetes, inflammation, cancer) and MitoCarta (mitochondrial genes) [48] and cross-referenced with genes significantly altered between HFD and SO-HFD. Venn diagrams were created using the online tool VENNY [49]. All RNA-seq data have been submitted to GEO, accession number GSE68360.

### Quantitative Real-Time PCR

Quantitative real-time PCR (qRT-PCR) was performed using the Bio-Rad-CFX96 Real-Time PCR Detection System (Bio-Rad, Hercules, CA, USA) to verify the relative expression of *Cidea* in the livers of mice fed Viv, HFD or SO-HFD for 16 or 35 weeks. Total RNA was extracted as described above and RT-PCR was performed using the QuantiTect Reverse Transcription Kit (Qiagen) for cDNA synthesis, followed by PCR using the QuantiFast SYBR Green PCR Kit (Qiagen). Thermal cycling conditions were 5 min at 95°C followed by 35 cycles of 10 s at 95°C and 30 s at 60°C. *Cidea* primers (forward: 5’-ATCACAACTGGCCTGGTTACG-3’; reverse: 5’-TACTACCCGGTGTCCATTTCT-3’) were gifts from Dr. Joseph Baur at University of Pennsylvania, Philadelphia. The relative expression level of *Cidea* was determined by normalizing to cyclophilin A (*Ppia*) expression as described previously [50].

### Global Metabolic Profiling

Livers from mice that had been fed Viv, HFD or SO-HFD for 16 weeks or 35 weeks were harvested, rinsed briefly in PBS, and immediately frozen in liquid nitrogen. Frozen liver samples (6–8 per treatment group) were shipped to Metabolon Inc (Durham, NC) where they were extracted and prepared for analysis using a previously described standard solvent extraction method [51]. The extracted samples were split into equal parts for analysis on the GC/MS and LC/MS/MS platforms. Also included were several technical replicate samples created from a homogeneous pool containing a small amount of all study samples. The analysis yielded a dataset comprising a total of 398 compounds of known identity (referred to as biochemicals). Metabolic pathways were visualized using the Cytoscape plugin in the Metabolync Portal (https://portal.metabolon.com)

### Statistical Analysis

Data are presented as means ± standard error of means. One-way ANOVA with Tukey’s post-hoc analysis (GraphPad Prism version 6 for Mac, GraphPad Software, La Jolla, CA USA) was used to test for differences between groups. Welch’s two-sample *t*-test and an estimate of the false discovery rate (*q*-value) were used for analyzing the metabolomics data. Student’s *t*-test was used when comparing two conditions. Statistical significance for all data was set at *P* ≤ 0.05 and approaching significance at 0.05 < *P* < 0.10.

## Results

### Soybean oil induces more weight gain and adiposity than fructose

In order to compare the effects of a diet enriched in soybean oil to one consisting primarily of saturated fat from coconut oil, and to examine the effects of fructose, we designed four isocaloric diets: high fat diet (HFD, 40 kcal% fat, primarily from coconut oil), soybean oil-enriched HFD (SO-HFD), fructose-enriched HFD (F-HFD) and fructose-enriched SO-HFD (F-SO-HFD) (Table 1). The total fat content in these diets is similar to the current American diet (37–39 kcal%) as is the amount of soybean oil, based on LA composition (~10 kcal%) (Table 1) [16,36,52]. Coconut oil, which consists mainly of saturated fats of chain length 12 to 18 (Table 2), was used as the primary source of fat as it is naturally low in LA and other PUFAs, whereas diets made from lard (which is typically used in rodent studies) can have variable amounts of PUFAs depending on what the animals have been fed [24,53,54]. Therefore, the use of coconut oil allowed us to study the metabolic effects of soybean oil in a saturated fat background, without affecting the final PUFA concentrations. The amount of fructose in our diets (25 kcal%) is comparable to human consumption and that used in rodent studies [55–58].

**Fig 1:**
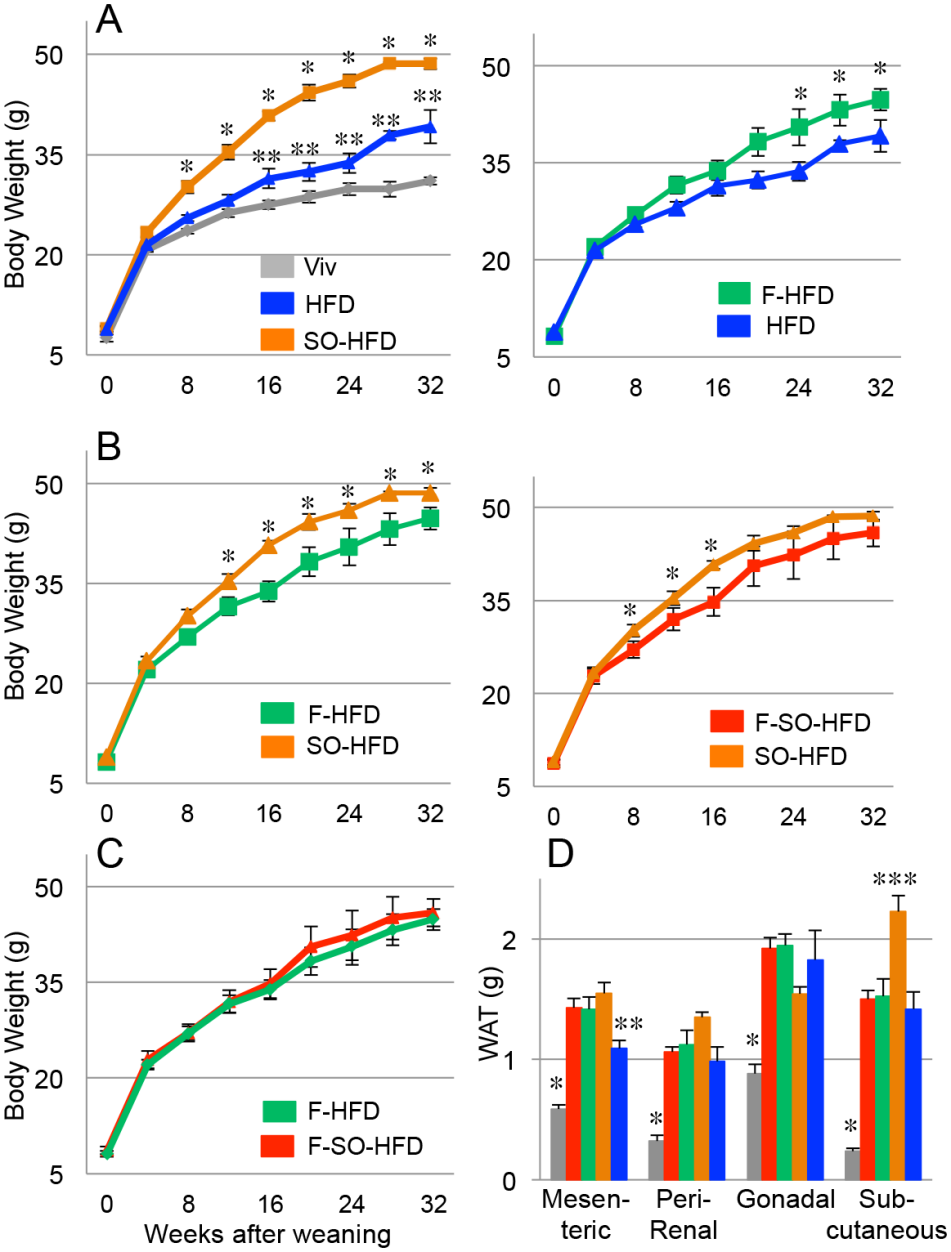
Soybean oil induces greater weight gain and adiposity than fructose. **A-C)** Average weekly body weights of male C57/BL6 mice started on the indicated diets at weaning. All diets are isocaloric with 40 kcal% total fat except Viv chow, which has 13.5 kcal% fat. HFD, high fat diet largely from coconut oil; SO-HFD, high soybean oil diet; SO-F-HFD, high soybean oil and high fructose diet; F-HFD, high fructose diet. N = 6–12. * Significantly higher than all others; ** HFD significantly higher than Viv. Significance is defined as P ≤ 0.05 by Student’s T-test. **D)** Average weight of different types of white adipose tissue. Diets are color coded as in **A-C**. * Significantly lower than all others; ** HFD significantly lower than SO-HFD; *** SO-HFD significantly higher than all others; N = 6–12 per diet. Significance is defined as P ≤ 0.05 by ANOVA with Tukey’s post-hoc analysis.

Male C57/BL6 mice at weaning were put on one of the four HFDs or a standard low fat, high fiber vivarium chow (Viv). Food intake did not differ significantly between the four HFDs (S1 Fig). The Viv chow-fed mice consumed more grams of food than the HFD-fed mice: this is to be expected since the chow is high in fiber and low in calories (3.36 kcal/gm) compared to the HFD (4.87 kcal/gm). Mice fed SO-HFD gained more weight and at a faster rate than mice fed HFD (Fig 1A left). Addition of fructose also increased body weight above HFD (Fig 1A right), although not as much as soybean oil (Fig 1B left). SO-HFD mice gained slightly more weight than F-SO-HFD mice, although this difference was significant only between weeks 8 and 16 (Fig 1B right). Weights of mice on the high fructose diets, irrespective of soybean oil content, did not differ significantly from each other (Fig 1C).

The amount of mesenteric and subcutaneous white adipose tissue (WAT) was significantly greater in SO-HFD than HFD mice; the amount of peri-renal fat was trending in the same direction. Fructose-fed mice (F-HFD and F-SO-HFD) had similar amounts of mesenteric fat and peri-renal fat as SO-HFD mice but lower amounts of subcutaneous WAT. SO-HFD mice had the lowest amount of gonadal WAT compared to the other three HFDs although the difference was not statistically significant (Fig 1D). The most notable difference was in the subcutaneous fat where SO-HFD was markedly greater than the other three HFDs (Fig 1D).

### Fructose increases liver/body weight ratio and synergizes with soybean oil to increase kidney weight; coconut oil increases spleen weight

**Fig 2:**
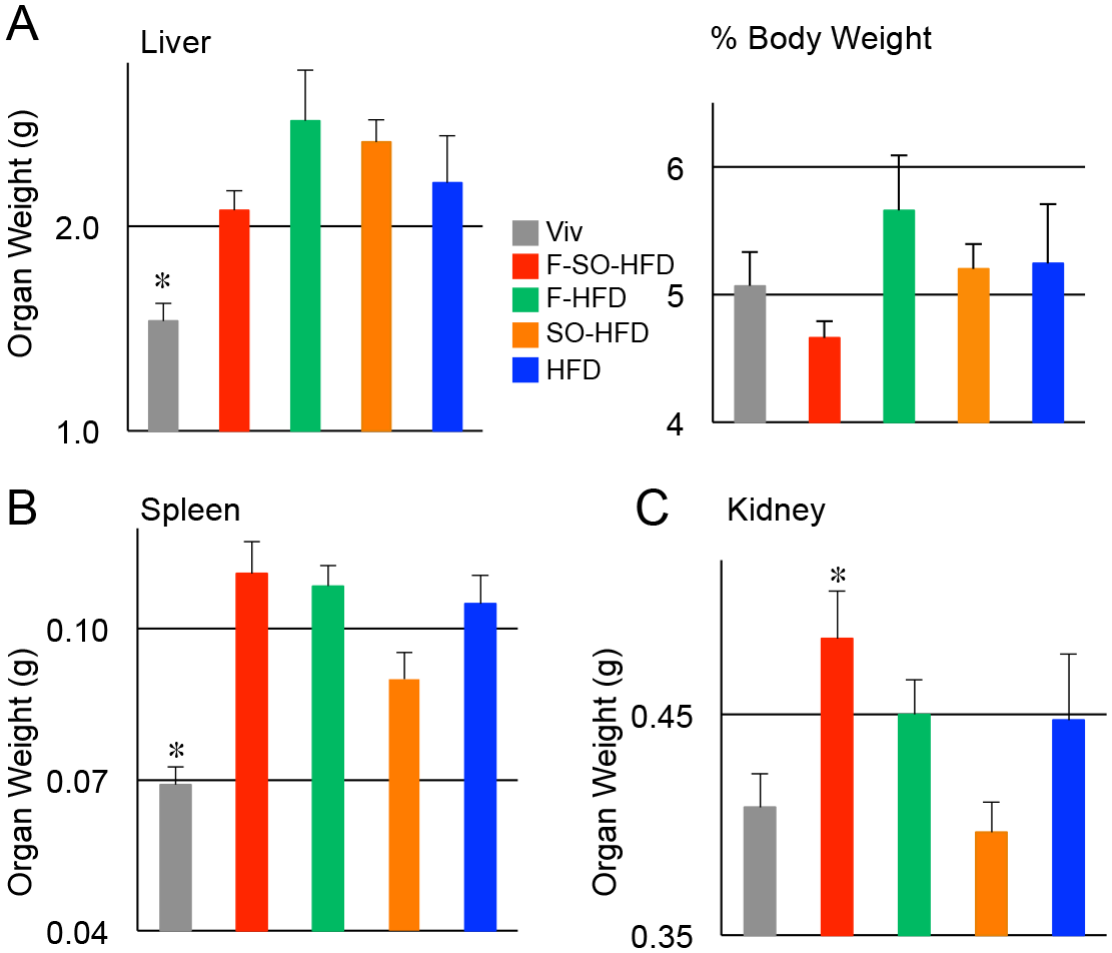
Fructose enhances liver/body weight ratio and kidney weight; soybean oil is protective in spleen and kidney. Average weight of organs harvested from C57/BL6 mice on the indicated diets as in Fig 1 for 35 weeks. **A) Left**, total liver weight. * Significantly lower than SO-HFD and F-HFD. **Right**, liver as percent of body weight. **B)** Spleen weights. * Significantly lower than all others except SO-HFD; **C)** Kidney weights (both combined). * Significantly higher than Viv and SO-HFD;. N = 6–12 per diet. Significance is defined as P ≤ 0.05 by ANOVA with Tukey’s post-hoc analysis.

Liver size is well known to adjust to body size. Mice fed F-HFD or SO-HFD had larger livers (by weight) at the time of harvest compared to the Viv chow fed mice (Fig 2A left). When normalized to body weight, F-HFD had the greatest liver-to-body weight ratio, while F-SO-HFD was the lowest, although the differences were not statistically significant (Fig 2A right).

Spleen weight was examined as a potential sign of infection, liver disease and inflammation. All four HFDs had elevated spleen weights compared to Viv, although the difference between SO-HFD and Viv was not statistically significant (Fig 2B). Kidneys showed a profile similar to spleen, although with larger differences between the diets: F-SO-HFD had the largest kidney weight while SO-HFD was similar to Viv (Fig 2C). These results suggest that the diet high in saturated fat from coconut oil may have a negative effect on the immune system and that soybean oil may counter those effects. In contrast, in the kidney there may be a synergistic effect between fructose and soybean oil.

### Intestinal length is shortened by all four HFDs

**Fig 3:**
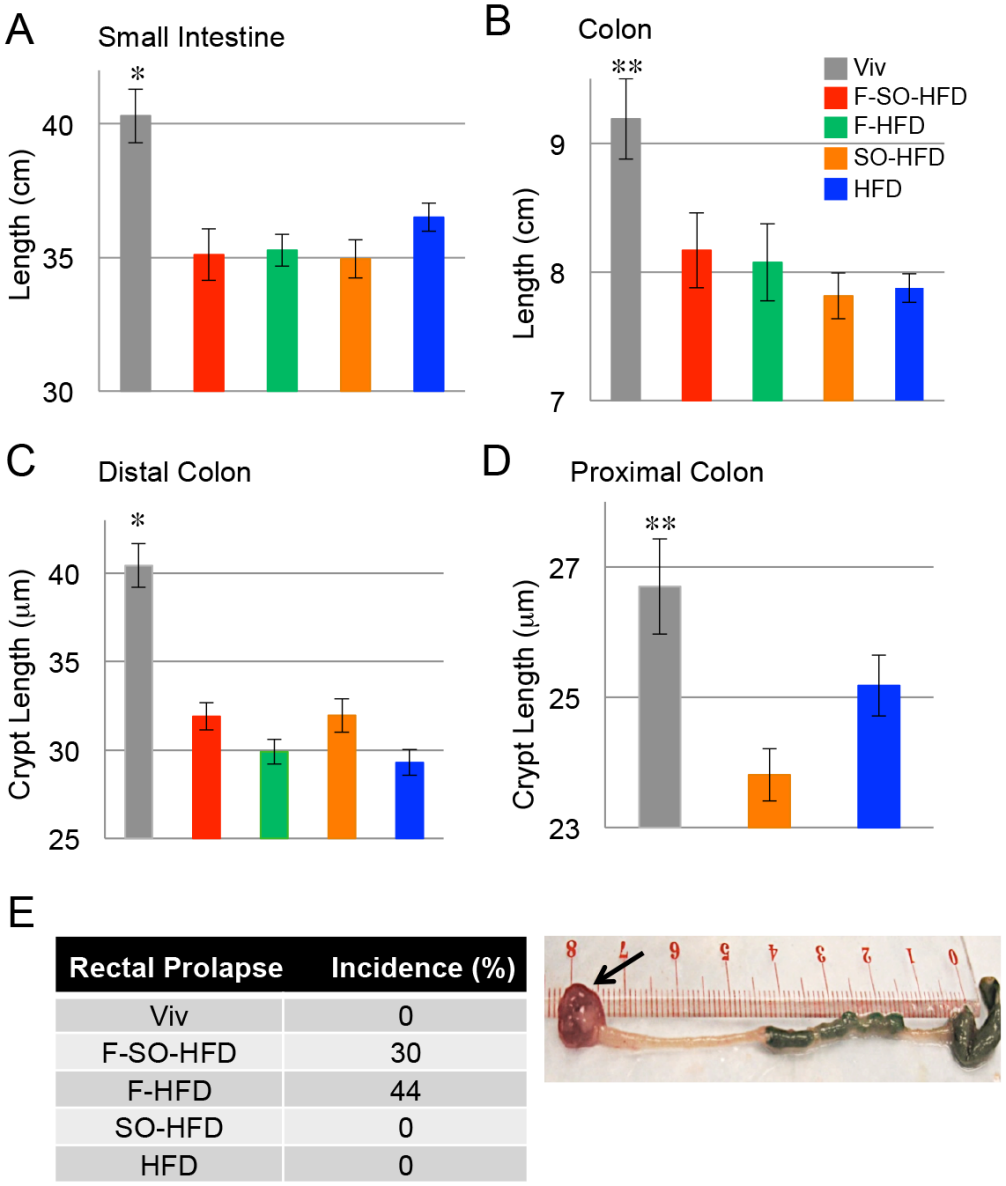
Soybean oil and fructose affect intestinal morphology. Length of small intestine **(A)** and colon **(B)** of C57/BL6 male mice on the indicated diets for 35 weeks. N = 6–12 per diet. * Significantly higher than all others. ** Significantly higher than SO-HFD and HFD. Average crypt length in the proximal **(C)** and distal **(D)** colon. N = 3–4 mice per group (up to 25 crypts measured/mouse). * Significantly higher than all others. ** Significantly higher than SO-HFD. Significance is defined as P ≤ 0.05 by ANOVA with Tukey’s post-hoc analysis. **E) Left**, incidence of rectal prolapse at 35 weeks, n = 12 per group. **Right**, representative image of rectal prolapse (arrow) in a mouse on F-HFD for 35 weeks.

Since the intestines play a major role in nutrient absorption [59], we examined their overall morphology. Mice on all four HFDs had significantly shorter small intestines compared to Viv-fed mice (Fig 3A and 3B). Colon length was also shorter in these mice although the difference was significant only between Viv versus SO-HFD or HFD. These effects are most likely due to the reduced amount of fiber in the HFD diets compared to the fiber-rich Viv chow as fiber is known to increase intestinal mass and crypt cell production [60]. Colonic inflammation and gross changes in colon morphology were not observed in the colons of any of the diet-treated mice (data not shown), although all four HFDs had decreased crypt length in the distal colon (Fig 3C). In contrast, in the proximal colon SO-HFD had a significantly shorter crypt length than Viv; crypt length was also shorter than in HFD although that difference was not significant (Fig 3D). It is known that crypt length and response of the colonic epithelial cells to external stimuli can vary along the length of the rodent intestine [61,62]. The most notable effect on the gut was that fructose dramatically increased the incidence of rectal prolapse to 30% in F-SO-HFD and 44% in F-HFD (Fig 3E). Taken together, these results indicate that soybean oil may protect against the effects of a diet high in saturated fat (and/or low in fiber) in the distal but not the proximal colon, and that fructose severely impacts the rectum.

### Soybean oil induces diabetes, glucose intolerance and insulin resistance (IR)

**Fig 4:**
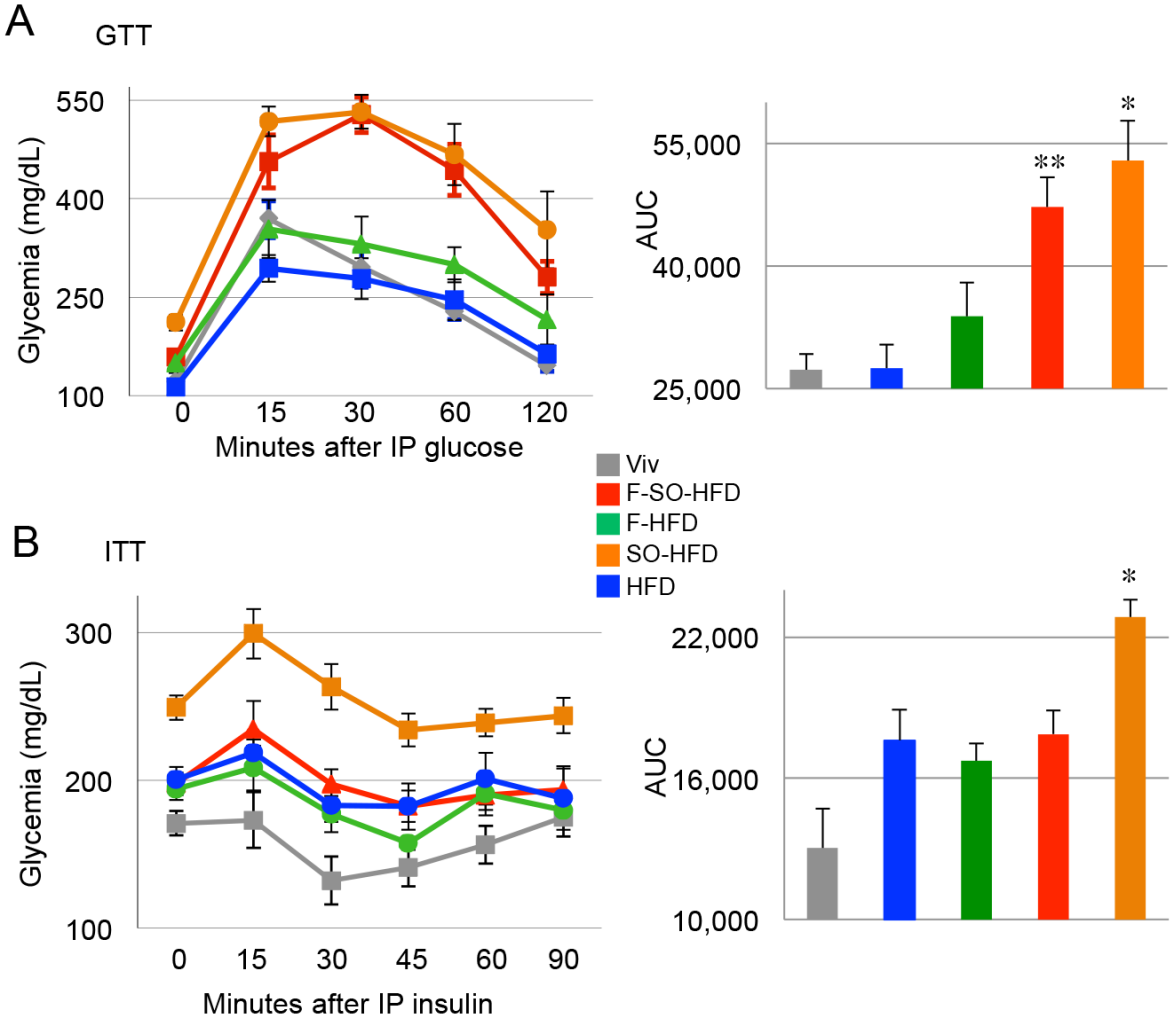
Soybean oil induces diabetes and insulin resistance (IR). **A)** Left panel: GTT assay of male mice on the indicated diets for 20 weeks. Right panel: Area under the curve (AUC) for GTT. * SO-HFD significantly higher than HFD, F-HFD and Viv. ** F-SO-HFD significantly higher than Viv and HFD. **B**) Left panel: ITT assay of mice on the indicated diets for 33 weeks. Right panel: Area under the curve (AUC) for ITT. * SO-HFD significantly higher than others. Significance is defined as P ≤ 0.05 by ANOVA with Tukey’s post-hoc analysis. N = 6–12 per group.

Since there is a considerable debate in the literature about whether HFCS in sodas or processed foods contributes to diabetes [33,63], we examined glucose tolerance and insulin sensitivity by GTT and ITT. To our surprise, we found that at 20 weeks the F-HFD did not cause diabetes (fasting blood glucose level > 200 mg/dL) whereas the SO-HFD did (Fig 4A). Furthermore, the F-HFD mice were just barely less tolerant to glucose than the Viv mice while the SO-HFD were extremely intolerant. Interestingly, the addition of fructose to SO-HFD (F-SO-HFD) actually slightly ameliorated the glucose intolerance of SO-HFD. Notably, the diet consisting primarily of coconut oil (HFD) did not show any diabetes or glucose intolerance at 20 weeks.

Even more striking were the results of the insulin tolerance test. At 33 weeks, the SO-HFD mice were the most insulin resistant and much more so than F-SO-HFD mice, which were indistinguishable from F-HFD and HFD (Fig 4B). All told, these results indicate that a moderately high fat diet of coconut oil, either in the presence or absence of fructose, does not induce significant diabetic symptoms (elevated fasting blood glucose and glucose intolerance) while isocaloric diets with soybean oil (either with or without fructose) do. Counter intuitively, our results also suggest that the addition of fructose to the diet may even protect against the IR caused by soybean oil.

### Soybean oil causes fatty liver and hepatocyte ballooning

**Fig 5:**
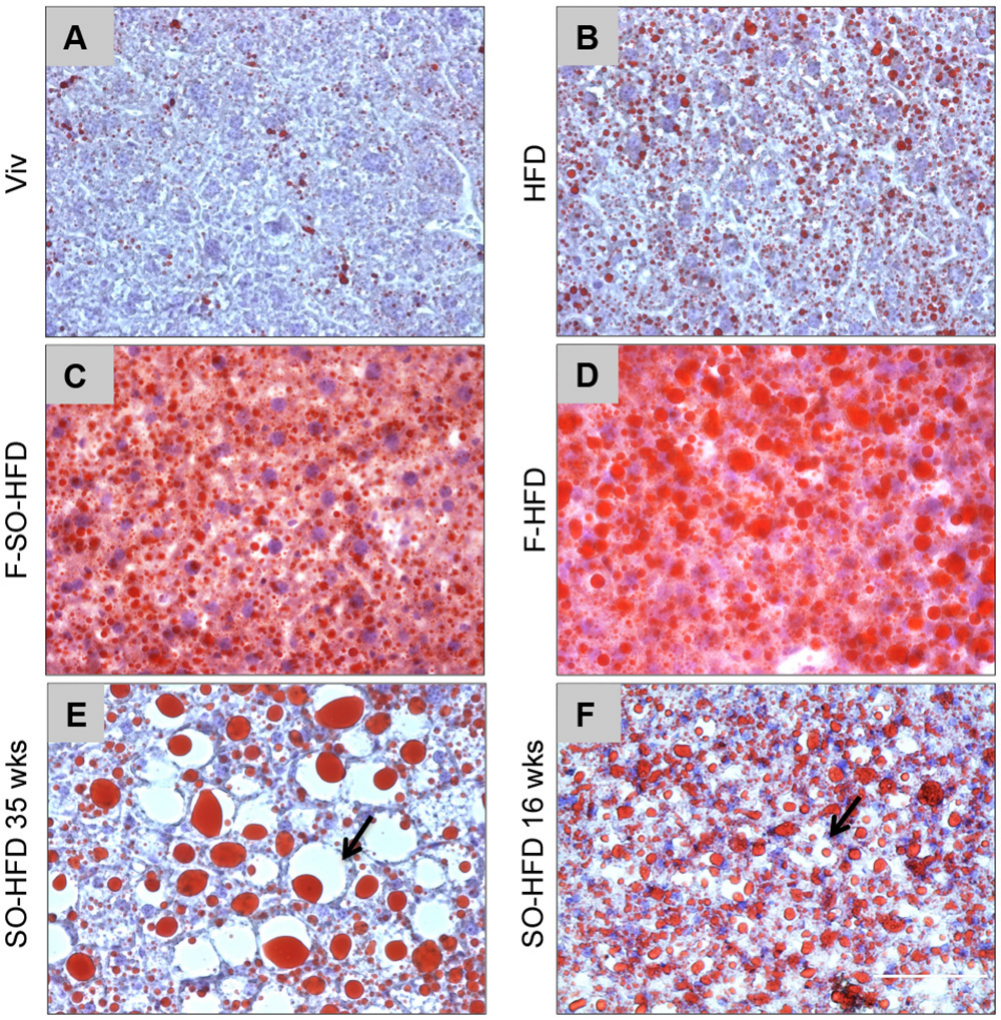
Soybean oil causes fatty liver and hepatic balloon injury. Representative Oil Red O staining for fatty liver in male mice on the various diets for 35 weeks **(A-E)** or 16 weeks **(F)**. **(E,F)** Arrows indicate ballooning injury in mice on SO-HFD. Scale bar (100 microns) is shown in **(F)**. Livers from 4–9 mice per group were examined: see S2 Fig for images from additional mice.

Since the liver is a major metabolic organ involved in lipid metabolism, we stained the mouse livers with Oil Red O (ORO) and observed fatty livers in both the soybean oil and fructose-fed mice although there were important differences between the two diets (Fig 5). While fructose caused excessive but typically fairly uniform fat deposition (Fig 5C and 5D), as has been observed previously [64,65], the SO-HFD livers had very large lipid droplets that were consistently accompanied by severe hepatocyte ballooning, suggesting potential liver damage (Fig 5E). The macrovesicular steatosis and balloon injury was also observed in livers of mice that had been on SO-HFD for just 16 weeks although the effects were not as large as at 35 weeks (Fig 5F). Despite the fatty livers and extensive tissue injury (in the case of SO-HFD mice), there was minimal liver fibrosis in all five diets (data not shown).

### Soybean oil causes a dysregulation of liver gene expression

**Fig 6:**
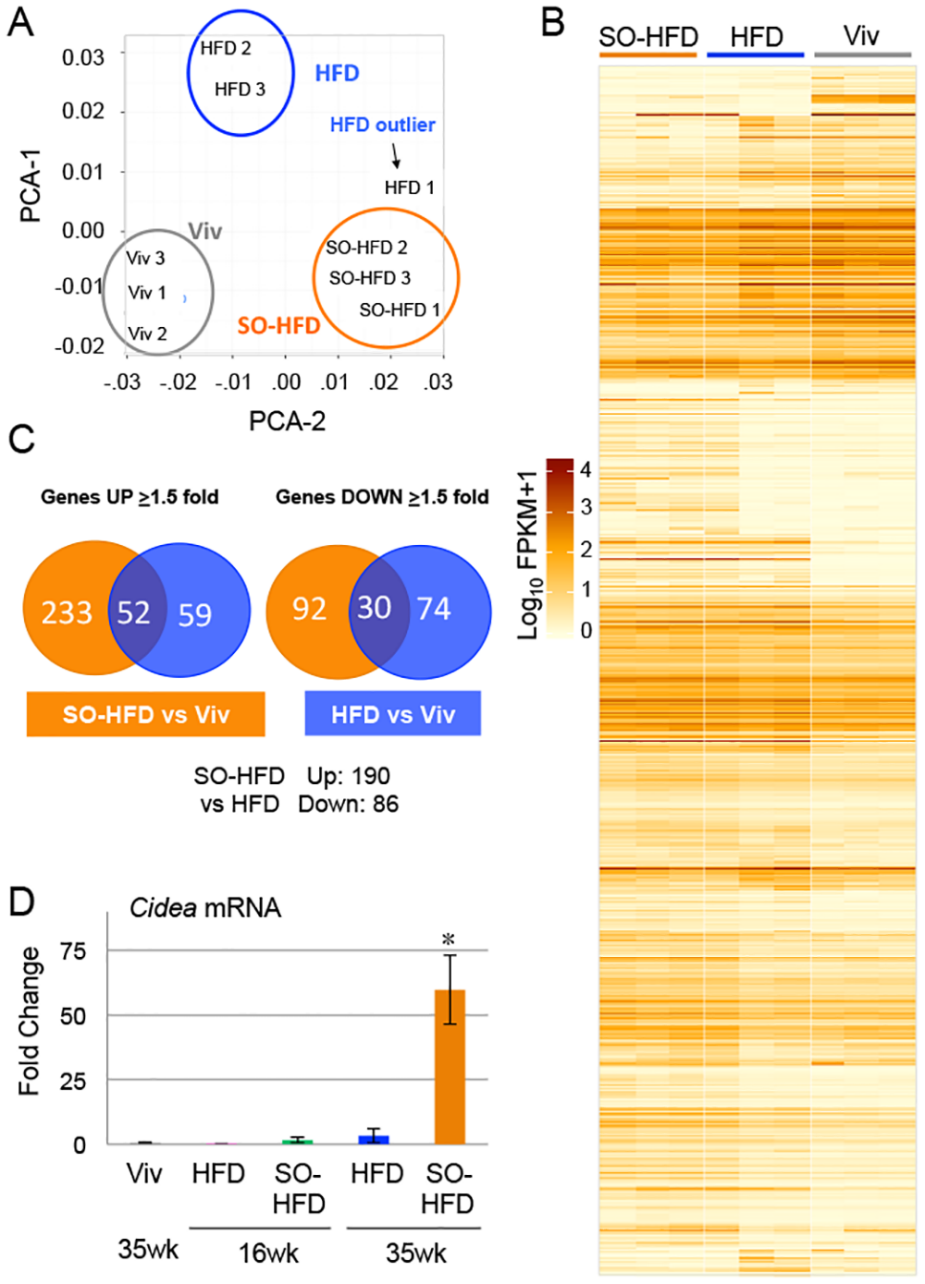
Soybean oil causes a distinct dysregulation of hepatic gene expression from coconut oil. **A)** Principle components analysis (PCA) of RNA-seq from livers of male mice fed Viv, HFD or SO-HFD for 35 weeks showing the three biological replicates clustering together for each diet except for one HFD outlier. **B)** Heat map showing differential gene expression between SO-HFD, HFD and Viv livers. **C)** Venn diagrams showing genes dysregulated in HFD or SO-HFD versus Viv. **D)** Increased Cidea mRNA expression confirmed by qPCR in the livers of mice fed SO-HFD compared to mice fed Viv or HFD for 16 or 35 weeks. * SO-HFD (35 weeks) is significantly higher than all others. Significance is defined as P ≤ 0.05 using a Student’s t-test. Three livers were assayed in triplicate for all conditions except Viv, for which two livers were analyzed. See S1 Dataset for raw cycle count (Cq) values obtained by qPCR for Cidea and cyclophilin A.

Since the SO-HFD mice exhibited the worst metabolic effects in terms of obesity, diabetes and IR, and since the livers of the SO-HFD mice had such a striking morphology, we performed RNA-seq on the livers of Viv, HFD and SO-HFD fed mice at 35 weeks. The three replicates for each diet clustered together with the exception of one outlier for HFD, which was closer to SO-HFD (Fig 6A and 6B). (The data from the HFD outlier was not included in the subsequent analyses.)

Both HFD and SO-HFD livers showed significant dysregulation of gene expression compared to Viv livers (Fig 6B and 6C). However, out of the ~120 known genes downregulated more than 1.5-fold (log2, 2.8-fold non log) in SO-HFD versus Viv, only 30 were also downregulated in HFD, suggesting that the type of dietary fat, in addition to the amount, has an impact on the liver, consistent with the morphological changes (Fig 5). There were almost three times as many upregulated genes in SO-HFD compared to HFD (285 versus 111, respectively), of which 52 were common to both diets. A direct comparison of SO-HFD to HFD showed 190 genes upregulated and 86 downregulated (See S1 Dataset for a complete list of the dysregulated genes)

*Cidea* (cell death-inducing DFFA-like effector a) was the most upregulated gene in SO-HFD (~120-fold versus Viv) and barely detectable in Viv and HFD livers (Fig 6D). Since *Cidea* has been implicated in dysregulated lipid metabolism [66] as well as the development of obesity, fatty liver and steatosis [67], we examined *Cidea* expression at 16 weeks by qPCR to determine whether it could be a causative factor in the soybean oil-induced metabolic effects. The results show that *Cidea* expression was not significantly upregulated at 16 weeks in SO-HFD (Fig 6D).

**Fig 7:**
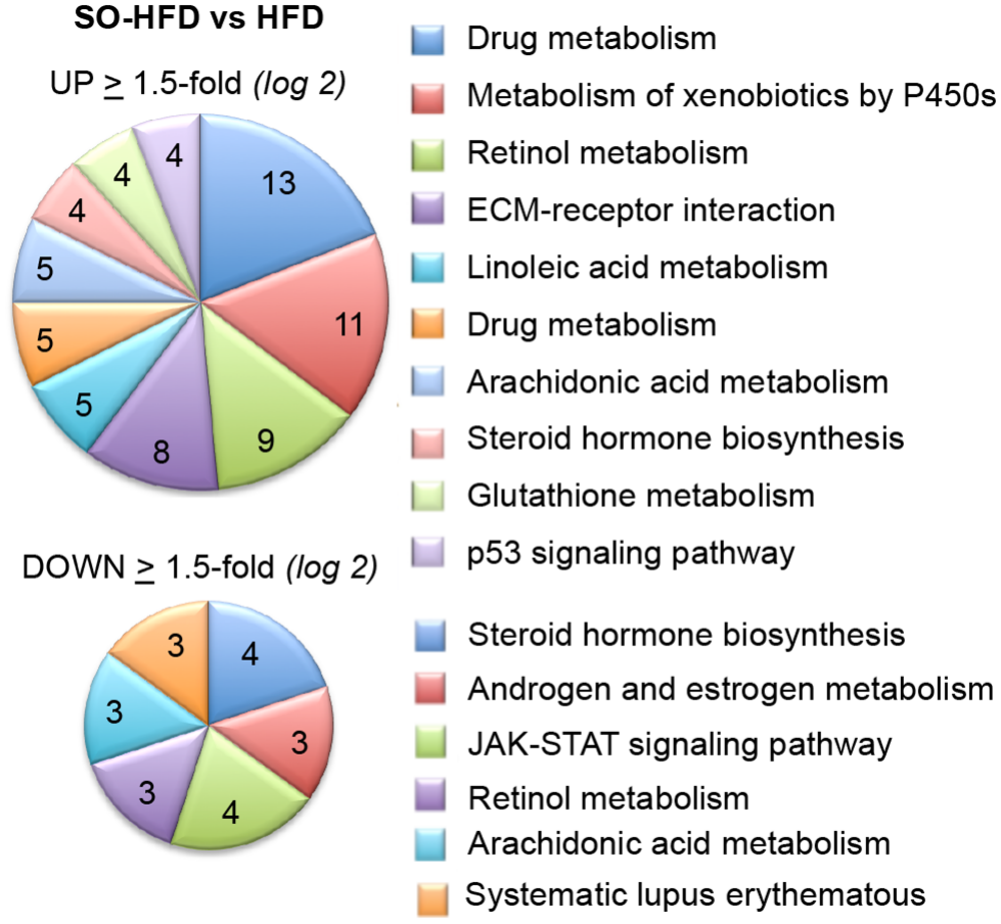
Gene ontology analysis of liver genes dysregulated by soybean oil. Functional annotation clustering of genes dysregulated ≥ 1.5-fold (log2) in SO-HFD versus HFD male mouse livers in RNA-seq. **Top**, upregulated genes; **Bottom**, downregulated genes.

Gene ontology showed, unexpectedly, that the category with the highest number of significantly upregulated genes between SO-HFD and HFD is that of xenobiotic and drug metabolism (29 genes total) (Fig 7).

**Fig 8:**
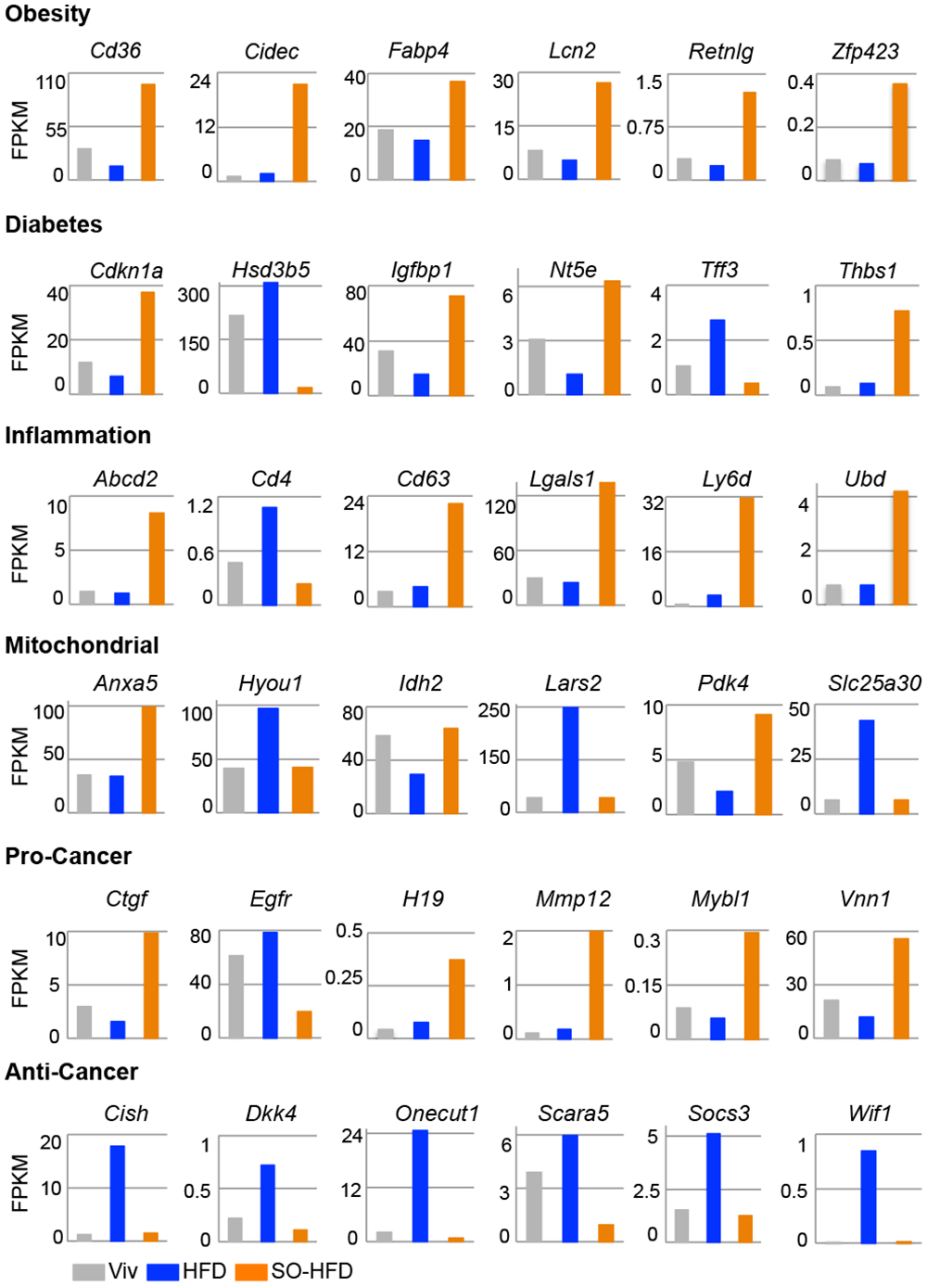
Select liver genes dysregulated in SO-HFD related to metabolic disease and cancer. Absolute expression levels from RNA-seq data in FPKM of dysregulated genes in the livers of Viv, HFD and SO-HFD fed male mice at 35 weeks. Shown are six representative genes for each category out of 13 obesity, 14 diabetes, 27 inflammation and 27 mitochondrial dysfunction and 31 cancer related genes identified by comparison with Pubmed Gene lists. Pro- and anti-cancer genes were curated manually. Some genes may belong to more than one category. SO-HFD values are significantly different (q-value ≤ 0.05) from both Viv and HFD for all genes except for *Hyou1*, *Idh2*, *Lars2*, *Sc25a30*, *Cish*, *Dkk4*, *Socs3* and *Wif1* where SO-HFD is significantly different only from HFD.

A number of the dysregulated genes were also found to be associated with one or more disease conditions such as obesity, diabetes, inflammation, mitochondrial dysfunction, and/or cancer (Fig 8), many of which also overlapped with liver disease (not shown). Most, but not all obesity, diabetes and inflammation promoting genes had elevated expression in SO-HFD versus HFD livers, as well as versus Viv. For example in the obesity category, the fatty acid translocase *Cd36*, which aids in free fatty acid (FFAs) uptake and contributes to hepatic steatosis [68] and fatty acid binding protein *Fabp4* that helps maintain hepatic metabolic balance and links diet induced obesity to insulin resistance [69,70] was increased in SO-HFD, while fatty acid binding protein 5 (*Fabp5*), which plays an important role in detoxifying FFAs and preventing lipid dysregulation [71], was decreased three-fold (S1 Dataset). Similarly, *Igfbp1*, an important regulator of insulin like growth factor 1 (IGF1) activity, showed an almost five-fold increase in SO-HFD versus HFD livers: increased hepatic expression of *Igfbp1* is associated with diabetes [72]. In the inflammation category, *Lgals1* (Galectin-1), an important immune response modulator and biomarker for hepatocellular carcinoma (HCC) [73–75] was also markedly increased in SO-HFD but not HFD, as were *Abcd2*, *Cd63*, *Ly6d* and *Ubd*.

Genes involved in mitochondrial function were more evenly split between elevated in HFD or SO-HFD. The most notable gene was *Pdk4*, which was elevated four-fold in SO-HFD compared to HFD and encodes a mitochondrial gene that plays an important role in the balance between glucose and fatty acid oxidation [76]. In contrast, *Hyou1 and Slc25a30 (KMCP1)* were expressed at higher levels in HFD compared to both SO-HFD and Viv. Since these genes are considered to be protective against oxidative damage [77–80], this suggests that coconut oil may be beneficial but that soybean oil diminishes the effect.

Genes in the cancer category showed a definite predominance of pro-proliferation genes upregulated in SO-HFD while anti-proliferative genes tended to be upregulated in HFD but not SO-HFD. This suggests that coconut oil may also be protective against liver cancer, though the protection may be nullified by soybean oil (Fig 8). (See S2 Dataset for a complete list of the genes in each category).

### Soybean oil causes a dysregulation of hepatic *Cyp* gene expression

**Fig 9:**
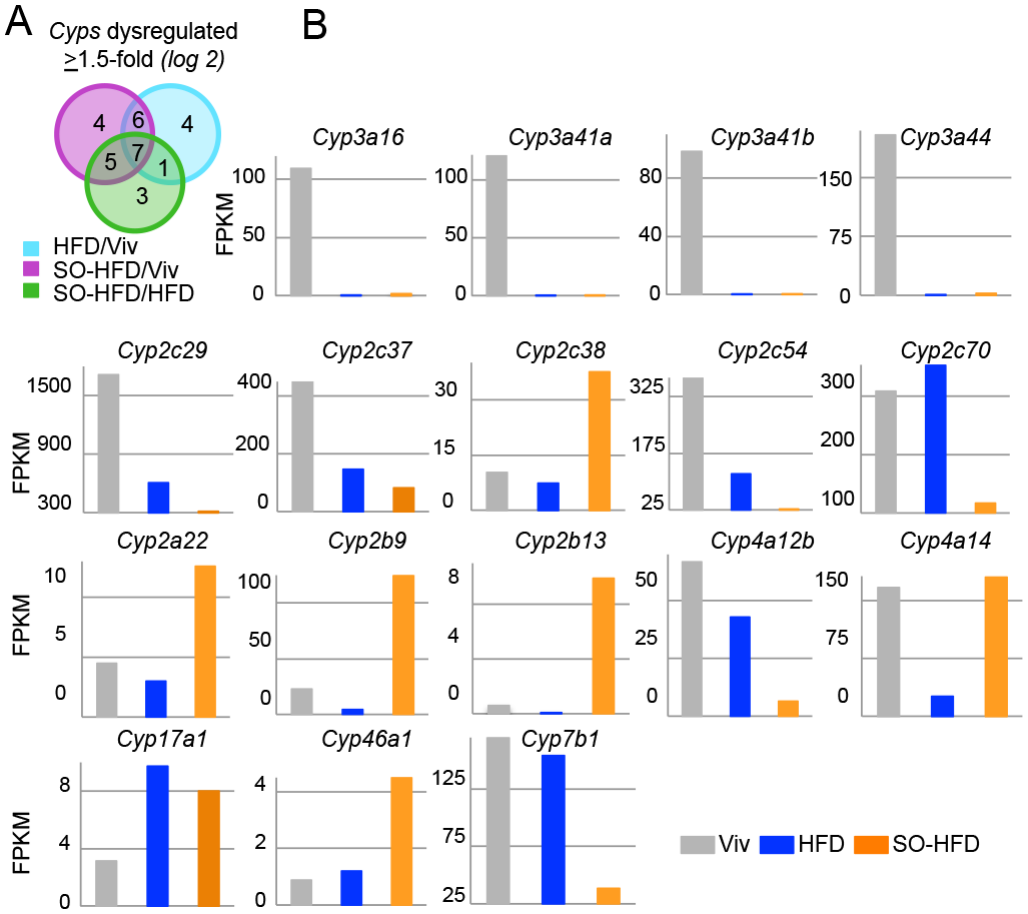
Dysregulation of *Cyp* genes in HFD and SO-HFD livers. **A)** Dysregulated (≥1.5-fold log2) *Cyp* genes in liver RNA-seq of male mice at 35 weeks. **B)** Absolute expression in FPKM of various *Cyp* genes as in Fig 8. SO-HFD is significantly different (q-value ≤ 0.05) from both Viv and HFD for all genes except *Cyp4a14* (SO-HFD not different from Viv) and *Cyp17a1* (SO-HFD not different from HFD).

The single most highly represented family of dysregulated genes (≥1.5-fold log2) was that of the cytochrome P450 (*Cyp*) genes (30 genes total) (Fig 9A). The most prevalent subfamilies were *Cyp2c* (nine genes total) and *Cyp3a* (six genes), followed by *Cyp2b* (three genes), and *Cyp4a* (three genes), all of which are in the LA or arachidonic acid (AA) metabolism pathways and most of which also metabolize steroids (Fig 9B). At first pass, this is consistent with soybean oil being highly enriched for LA and AA being a metabolite of LA. However, paradoxically, genes that encode P450 enzymes and use LA as a substrate, such as *Cyp2c54*, were downregulated even more in SO-HFD than HFD. Interestingly, *Cyp3a* family members (*Cyp3a16*, *Cyp3a41a*, *Cyp3a41b*, *Cyp3a44*), which play an important role in drug metabolism, are significantly downregulated in HFD livers but somewhat less downregulated in SO-HFD (Fig 9B and S3 Dataset). In contrast, a number of other *Cyps* (e.g., *Cyp2a22*, *2b9*, *2b13*, *2c38*, *4a14*, *46a1*) are uniquely upregulated in SO-HFD. It is possible that downregulation of the *Cyp3a* family triggers a compensatory upregulation of these other *Cyp* genes, as was shown previously in *Cyp3a^-/-^* mouse studies [81]. *Cyp17a1*, an important enzyme in the steroidogenic pathway, is increased by both SO-HFD and HFD, while two other *Cyp* genes involved in cholesterol and bile acid metabolism (*Cyp46a1* and *Cyp7b1*) show opposite effects in terms of activation in SO-HFD or HFD livers. Importantly, the expression of *Cyp7b1*, which is involved in the conversion of cholesterol to bile acids, is greatly reduced in SO-HFD but not HFD. This could result in lower levels of bile acids, which are known to play anti-obesogenic roles [82,83]. (See S3 Dataset for a complete list of significantly dysergulated *Cyp* genes.)

### Soybean oil increases accumulation of fatty acid metabolites in the liver

To examine changes in hepatic metabolites, we performed global metabolic profiling of the livers from mice fed Viv, HFD or SO-HFD for either 16 or 35 weeks. The analysis identified 398 named biochemicals and revealed a largely similar profile of up- and downregulated metabolites in HFD and SO-HFD versus Viv at 35 weeks (S3 Fig). There were more differences at 16 weeks on the diets especially in the category of lysolipids (S3 Fig). There were four lysolipids (1-linoleoylglycerophosphoinositol, 1-arachidonoylglycerophosphoinositol, 2-arachidonoylglycerophosphoinositol and 2-stearoyloylglycerophosphoinositol) that were up significantly at 16 weeks, but this difference was lost by 35 weeks. The metabolites that changed in a temporal fashion (35 versus 16 weeks) were also different in HFD and SO-HFD (e.g., long chain fatty acids and lysolipids) (S3 Fig). (See S4 Dataset for the complete metabolomics dataset.)

**Fig 10:**
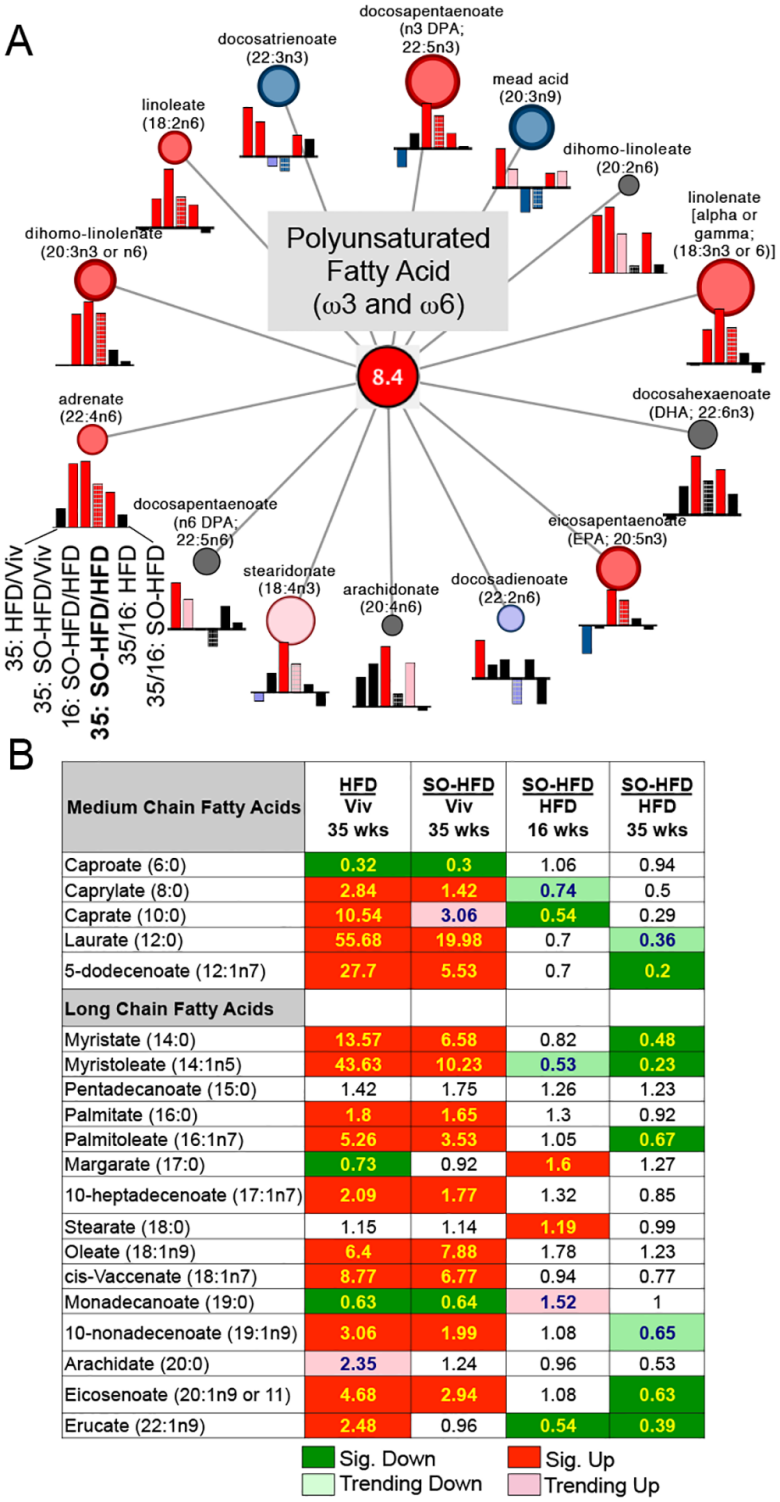
PUFA metabolites are enriched in SO-HFD versus HFD at 35 weeks. **A)** Cytoscape visualization of PUFA pathway enrichment (overall 8.4-fold) in SO-HFD versus HFD at 35 weeks. Circles indicate fold enrichment for SO-HFD versus HFD at 35 weeks (bold): red or pink, significantly (P ≤ 0.05) or trending significantly (P ≤ 0.1) up; blue or purple, significantly or trending significantly down; black, no change. Size of circles depicts relative fold change. Bar graphs below each metabolite show relative change for other comparisons as noted. **B)** Table showing fold change in medium chain and long chain fatty acids for the indicated comparisons. Significance for all data was set at P ≤ 0.05 and approaching significance at 0.05 < P < 0.10 as determined by Welch’s two-sample t-test.

Since the most prominent difference between SO-HFD and HFD was the category of PUFAs (ω3 and ω6) (S3 Fig), we looked more closely at individual PUFAs with ≥18 carbons and found that roughly half were elevated at 16 and 35 weeks; the only exceptions were docosatrienoate (22:3ω-3), mead acid (20:3 ω-9), docosadienoate (22:2ω-6), which were downregulated (Fig 10A). This increase in PUFAs is expected, as most of these are metabolites of LA, which is enriched in SO-HFD. The decrease in mead acid is also consistent with it being an indicator of essential fatty acid (EFA) deficiency [84]. Also elevated at 35 weeks in SO-HFD were α-linolenic acid (LNA, 18:3ω3 or 6) and eicopentaenoic acid (EPA, 20:5**ω**3), which should have a beneficial effect [85,86]. The increase in LNA and EPA is not surprising given that soybean oil has more of both the ω3 and ω6 PUFAs than coconut oil.

In contrast to PUFAs, most saturated and mono unsaturated medium (5–12 carbons) and long chain free fatty acids (FFAs) (≥14 carbons), while elevated at 35 wks in both HFD and SO-HFD relative to Viv, were reduced in SO-HFD versus HFD at both 16 and 35 weeks (Fig 10B). This is likely due to the replacement of some of the coconut oil, which is high in saturated fats, with soybean oil, causing a relative decrease in hepatic concentrations of beneficial medium chain triglycerides such as capric acid (C 10:0) and lauric acid (C 12: 0). Both capric and lauric acid have been shown to cause a decrease in adiposity, increased insulin secretion and improved serum lipid profile [87–89].

### Differential effects of HFD and SO-HFD on inflammatory metabolites, anti-oxidants and other metabolites in the liver

**Fig 11:**
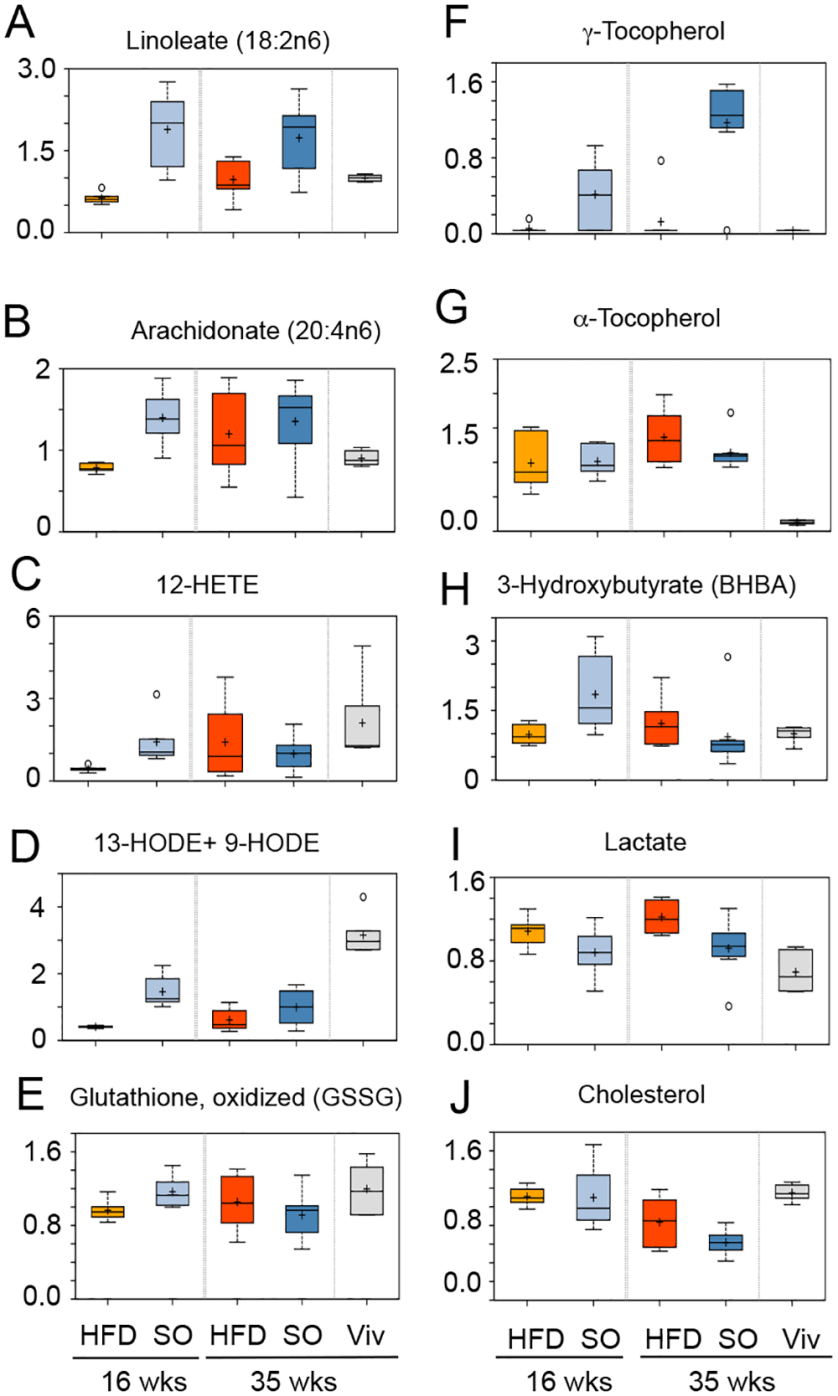
Hepatic metabolite levels change with diet and over time. Box-plots of liver metabolomics data from male mice fed the indicated diets for 16 or 35 weeks showing changes in levels of **A)** Linoleic acid, **B)** Arachidonic acid, **C)** 12-HETE, **D)** 13 HODE +9 HODE, **E)** Glutathione, oxidized, **F)** γ-Tocopherol, **G)** α-Tocopherol, **H)** 3-Hydroxybutyrate, **I)** Lactate, and **J)** Cholesterol. At 16 weeks SO-HFD is significantly different from HFD for all metabolites except γ-tocopherol, lactate and cholesterol. At 35 weeks there is no statistical difference between SO-HFD and HFD except for linoleic acid, γ-tocopherol and lactate; cholesterol is approaching significance. Significance, P ≤ 0.05; approaching significance, 0.05 < P < 0.10 by Welch’s two sample t-test. (See S4 Dataset for additional box plots.)

Not surprisingly, levels of LA and its metabolite AA were significantly increased in SO-HFD versus HFD and Viv at 16 weeks (Fig 11A and 11B). Interestingly, though, the levels of both LA and AA increased in HFD from 16 to 35 weeks but not in SO-HFD. These trends are consistent with the body’s tendency to accumulate and store LA [90], an essential fatty acid [40], and suggest that there may be an upper limit on that storage capacity, at least in the liver. The trend is also consistent with the RNA-seq results in which one HFD outlier was more similar to SO-HFD than the other two HFD samples. This could suggest that HFD and SO-HFD are on a similar metabolic trajectory, although the HFD mice do not reach the same level of adiposity, diabetes, IR or fatty liver as the SO-HFD mice, at least within 35 weeks on the diet.

Interestingly, the pro-inflammatory eicosanoid 12-HETE (an AA metabolite) and the marker of lipid peroxidation 13-HODE+9-HODE (LA metabolite) [91–93] were significantly decreased in HFD versus Viv at both 16 and 35 weeks, suggesting that coconut oil may be protective against inflammation (Fig 11C and 11D). The lower levels of these metabolites could be due to decreased expression of *Cyp2c54* (Fig 9B), which is known to metabolize AA and LA to HETEs and HODEs [92–95]. Interestingly, 12-HETE, 13-HODE+9-HODE and *Cyp2c54* were also all reduced in SO-HFD, although the metabolites were somewhat higher than in HFD: this could be due to the higher levels of LA in the soybean oil.

At 16 weeks there were significantly higher levels of oxidized glutathione (GSSG) in SO-HFD versus HFD but at 35 weeks the levels in SO-HFD were lower than those in HFD (Fig 11E). Since glutathione is a major anti-oxidant in the liver, this suggests a temporal effect on oxidation that is diet-dependent. This effect could be due to γ-tocopherol, which was highly elevated in SO-HFD at 16 weeks and even more so at 35 weeks (Fig 11F): γ-tocopherol, a form of Vitamin E, is a potent anti-oxidant and enriched in soybean oil [96]. In contrast to γ-tocopherol, α-tocopherol, was elevated in all four HFD samples with no enrichment in SO-HFD. While α-tocopherol is the most dominant form of Vitamin E in the body, and the best studied [97], there are reports that γ-tocopherol may have more potent anti-inflammatory and anti-cancer properties [98,99]. All told, these results show that while coconut oil is protective in terms of reducing levels of certain pro-inflammatory markers, soybean oil provides an additional source of anti-oxidants in the form of γ-tocopherol.

Elevated levels of the ketone body 3-hydroxybutyrate (BHBA) in SO-HFD versus HFD were noted at 16 but not 35 weeks (Fig 11H). Ketones are produced in the liver from excess acetyl-CoA, which is typically generated through fatty acid oxidation, suggesting altered lipid metabolism over time. In contrast, lactate levels were lower in SO-HFD compared to HFD at both 16 and 35 weeks (Fig 11I), suggesting reduced glucose utilization at both time points, which might be related to the higher blood sugar levels observed at 20 weeks (Fig 4A). Cholesterol was reduced at 35 weeks in both the HFD and SO-HFD livers compared to Viv **(** Fig 11J), with SO-HFD having the greatest effect.

## Discussion

There is currently considerable debate in both the scientific literature as well as the lay press as to which components of the American diet are the most obesogenic. Since diet studies in humans involve a large number of variables, most of which cannot be properly controlled, in this study we used mice and precisely defined isocaloric diets to compare the metabolic effects of saturated fat from coconut oil, unsaturated fats from soybean oil and fructose. To our knowledge, this is the first study not only to compare the effects of these three dietary factors in mice, but also to perform genome-wide expression profiling and metabolomics analysis of livers from animals fed a soybean-oil enriched diet. Our results indicate that, contrary to expectation, PUFA-rich soybean oil is more obesogenic and diabetogenic than coconut oil which consists of primarily saturated fat. They also show that fructose is less obesogenic than soybean oil and reveal a striking fatty liver morphology induced by soybean oil as well as a global dysregulation of *Cyp* genes and disease-associated genes and metabolites in the liver. These effects in the mouse liver could be clinically relevant as NAFLD, a component of the Metabolic Syndrome, is estimated to be present in 20–30% of adults in the U.S. and 3–10% of children [100,101].

### Soybean Oil versus Coconut Oil and Fructose

The increase in obesity in the U.S. over the last half-century coincides with a shift in the dietary preference away from saturated fats from animal products and toward plant-based unsaturated fats [16]. That shift was due in large part to the results of the Seven Countries Study, which was published in 1970 and implicated saturated fats as the causal factor for cardiovascular disease [6,102]. At the same time, soybeans were being promoted for cultivation and started to receive considerable federal subsidies [16,103]. As a consequence, soybean oil soon became the oil of choice for domestic as well as commercial cooking purposes in the U.S. [16,18]. The results presented here suggest that this dietary shift, while perhaps beneficial for cardiac health, may have aggravated other problems, such as obesity, diabetes, glucose intolerance, IR and fatty liver. Furthermore, our results show that soybean oil is able to induce these negative metabolic effects even in the context of coconut oil, which is rich in medium-chain triglycerides (MCTs) which have been shown to be anti-obesogenic, anti-inflammatory and insulin sensitizing [15,104,105].

Fructose consumption in the U.S. is also at an all time high [55,106–108]; its contribution to the obesity epidemic is even more hotly debated [109–112]. We show here that in mice dietary fructose does in fact induce obesity but less so than soybean oil. However, it did not induce diabetes or IR (Figs 1 and 4) although it may have a synergistic effect with soybean oil in the kidney (Fig 2C). Fructose caused excess lipid accumulation in the liver, as anticipated from human studies linking fructose consumption to NAFLD [113,114] as well as numerous mouse studies [115,116] (Fig 5). Finally, perhaps the most striking effect of the fructose-enriched diets was the high incidence of prolapsed rectums (Fig 3E), which is part of the disease activity index for inflammatory bowel disease (IBD), a disease that is on the rise [117] and considered to be a precursor for colon cancer [118]. Reports that a fructose-free diet can relieve gastrointestinal problems in humans [119] seem well founded.

### Hepatic lipid metabolism

Perhaps one of the most striking results from this study is the extent of hepatocyte ballooning and size of lipid droplets in the SO-HFD livers. Others have also noted that in rats a diet enriched in soybean oil leads to greater hepatic lipid accumulation than either lard or fish oil and concluded it was due to suppression of *Acox*, which encodes the enzyme responsible for the first and rate-limiting step in fatty acid oxidation [120,121]. While we did not observe a dysregulation of *Acox* in our mouse livers, we did observe an elevated expression of both *Acot1* and *Acot2*, as well as *Cd36 (a fatty acid transporter)*, in SO-HFD versus HFD, all of which could lead to hepatic lipid accumulation. We also saw a dysregulation of a number of mitochondrial genes (Fig 8). One of these genes, *Pdk4*, which was upregulated in SO-HFD livers, is known to inhibit the pyruvate dehydrogenase complex that links the TCA cycle with glucose and fatty acid metabolism. Repression of the pyruvate dehydrogenase complex shifts the balance towards gluconeogenesis which could result in hyperglycemia [76]. Consistent with our findings are reports that *PDK4* expression is increased in diabetics [122] and that *Pdk4* ^-/-^ mice are resistant to HFD-induced hepatic steatosis and are more glucose tolerant [123,124]. Thus *Pdk4* upregulation may be a contributing factor to both lipid accumulation in the liver and the development of diabetes and glucose intolerance in SO-HFD mice.

Lipid droplet formation is regulated by a number of proteins such as those in the PAT (perilipin, adipophilin, and the tail-interacting protein of 47 kDa, TIP47) and CIDE families [66,125–128]. The most highly up-regulated gene in the SO-HFD livers was *Cidea* (Fig 6D); *Cidec* and *Plin4*, a PAT family member, were also up regulated in SO-HFD versus HFD (S1 Dataset). All three genes have been associated with obesity [129], and *Cidea* and *Cidec* deficient mice are resistant to diet-induced obesity [130,131], suggesting they may play a causal role. However, in our experiments, *Cidea* was not greatly elevated at 16 weeks when body weight was already significantly different in SO-HFD versus HFD (Figs 1A and 6D), suggesting it might not be a driver of obesity in our diets. Nonetheless, CIDEA, along with CIDEC (and possibly PLIN4), could play a role in the formation of the huge lipid droplets seen in SO-HFD livers at 35 weeks as all three proteins (or related family members) have been shown to fuse together small lipid droplets to form larger ones [127,132]. Since the size of lipid droplets is dependent upon both their lipid and protein components, this could explain the remarkable difference in lipid droplet size between the various diets.

Fructose is known to increase *de novo* lipogenesis (and production of saturated fats) and cause fatty liver [55]; consistent with this, our results show that the F-HFD and F-SO-HFD mice also had excess fat in their livers (Fig 5D). Notably though, the size of the lipid droplets in F-SO-HFD was much smaller than SO-HFD, suggesting that the change in the FA profile of the liver by fructose [133] may affect the composition of the lipid droplets and hence their size. It remains to be determined whether there are any lipid binding proteins dysregulated in the fructose diets.

### Dysregulation of hepatic gene expression

Consistent with the striking change in liver morphology was the large-scale dysregulation of genes in the livers of SO-HFD mice (Figs 6–9). In addition to genes associated with obesity, diabetes, inflammation and mitochondrial function, 31 genes associated with cancer were also dysregulated (Fig 8, S2 Dataset). Several cancer-promoting genes were uperegulated (*Ctgf*, *H19*, *Mmp12*, *Mybl1*, *Vnn1*) while cancer inhibiting genes (*Cish*, *Dkk4*, *Onecut1*, *Scara5*, *Socs3*, *Wif1*) were suppressed. For example, *H19* is a long non coding RNA upregulated by the proto-oncogene MYC, elevated in a wide range of cancers and associated with risk factors such as exposure to carcinogens [134]. Some of the genes in the non cancer category are also associated with cancer. In contrast, one cancer-inhibiting gene p21/WAF1 (*Cdkn1a*) was up-regulated in SO-HFD but it is also considered to be an epiobesogene [135] and is linked to hepatic steatosis and liver dysfunction in offspring born to mothers fed a high fat diet [136]. While p21 is best known as a target of tumor suppressor p53 and an inhibitor of the cell cycle [137,138], it has also been reported to exhibit oncogenic activity [139]. It should be noted, however, that we did not observe any upregulation of the major drivers of hepatocellular carcinoma (HCC) (MYC, IL6, β-catenin) [140] in SO-HFD, nor any signs of tumors or neoplastic lesions. Furthermore, the signature fatty acid profile for HCC is one in which there is a decrease in long chain PUFAs, including LA, which is the opposite of what we observed in our metabolomics data [141,142]. Nonetheless, the dysregulation of cancer genes in SO-HFD-fed mice suggests that, in addition to excess body weight, which is itself a risk factor for liver cancer [143], a diet enriched in soybean oil might increase one’s susceptibility to liver cancer. Liver cancer incidence, just like obesity, has been steadily increasing over the last few decades [144,145].

At least 30 *Cyp* genes were dysergulated in SO-HFD and/or HFD, the majority of which are involved in LA, AA or steroid metabolism (Fig 9). A major component of soybean oil (54–70%) is LA, and AA is a metabolite of LA, which would explain their elevated levels in SO-HFD (Fig 10). Recently, Alvheim et al. [146] showed that increasing dietary LA from 1% to 8% significantly increased AA levels in liver and erythrocytes of mice, consistent with our data. (They also observed an increase in food intake and attributed the changes to increased levels of liver endocannabinoids, 2-arachidonoylglycerol (2-AG) and anandamide (AEA), neither of which we observed (S1 Fig and S4 Dataset)). Others have reported that LA does not increase AA levels in plasma and erythrocytes [147] but liver was not examined in those studies.

In terms of steroid metabolism, expression of the cholesterol 24-hydroxylase gene, *Cyp46a1*, was significantly increased in the SO-HFD livers compared to HFD. This is consistent with lower levels of cholesterol in SO-HFD livers (Fig 11). While the level of cholesterol in the plasma remains to be determined, this finding is consistent with the established beneficial effects of PUFAs on coronary arteries [148,149], as well as the dietary recommendation of LA for people with high cholesterol [10,150].

In addition to fatty acids and steroids, cytochrome P450 enzymes metabolize drugs and xenobiotics. The Cyp3a family, for example, metabolizes erythromycin, tamoxifen, codeine and morphine; while the Cyp2b and Cyp2c families are involved in cyclophosphamide and ifosfamide metabolism [151]. While for the *Cyp3a* genes, the largest decrease was between Viv and HFD or SO-HFD (Fig 9), there was also a significant decrease between HFD and SO-HFD, underscoring the notion that different diets might affect ones’ ability to metabolize drugs and/or xenobiotics.

The dysregulation of *Cyp* genes in SO-HFD can explain many of the differences in metabolites, but the question remains as to what alters the expression of the *Cyp* (and other) genes. While alterations in many genes, such as *Cidea*, could be secondary effects due to the accumulation of lipids in the liver, there must be some initiating events/factors. While identifying those factors is beyond the scope of the current study, some speculation may be informative. LA is the ligand for nuclear receptors PPARα, PPARγ and HNF4α [50,152,153]. PPARγ is associated with obesity [154] while decreased HNF4α is associated with fatty liver in mice [155] and diabetes in humans [156]. Many of the *Cyp* genes, *Cidec*, *Pdk4*, *Vnn1*, *Igfbp1*, *Acot2*, *Rgs3* and *Tceal8* and other dysregulated genes in SO-HFD compared to HFD are HNF4α target genes [157–161]. Likewise, PPARγ is known to regulate *Cd36*, *Fabp5* and *Retnlg* [162–164]. While the level of HNF4α and PPARγ RNA was not significantly altered in SO-HFD, LA could still be affecting HNF4α and PPARγ function.

## Concluding Remarks

While this study was in progress, two groups published papers with results similar to ours—namely, that a high fat diet supplemented with oils high in LA leads to obesity and fatty liver [24,53,146]. Other studies have also shown that dietary LA can cause adiposity in humans [165,166] and lead to hyperglycemia as well as obesity in mice [19,167]. Nonetheless, it should be noted that neither our study nor the others looked specifically at LA but rather at LA-enriched oils, leaving open the possibility that, while LA might be regulating the expression of certain genes via nuclear receptors, a component of the oils in addition to LA could be involved in one or more of the observed metabolic effects. Regardless of which components in soybean oil are responsible for those effects, its increasing use both in the U.S. and worldwide [16,168] warrants a detailed understanding of its effect on our health.

## Supporting Information

S1 Dataset Differentially regulated genes in SO-HFD, HFD and Viv livers. Significantly dysregulated genes (*P* < 0.05 and *q* < 0.1) from liver RNA-seq of male C57/BL6 mice fed SO-HFD, HFD or Viv chow for 35 weeks. Data are divided into four tabs: “HFD v Viv,” “SO-HFD v Viv,” “SO-HFD v HFD” and “Cidea Ppia Cq counts”. The first three sheets have the average FPKM values (and fold change) from RNA-Seq for three biological replicates per diet, except for HFD that had one outlier removed. The “Cidea Ppia Cq counts” sheet has the raw Cq values for Cidea and cyclophilin A obtained by qPCR.
(XLSX)

S2 Dataset Disease-related genes dysregulated in RNA-seq of SO-HFD versus HFD livers. Dysregulated genes (1.5-log(2) fold change) in SO-HFD versus HFD livers found by searching Pubmed Genes for “obesity,” “diabetes,” “inflammation” and “cancer.” Mitochondrial genes are from Mitocarta.
(DOCX)

S3 Dataset Differentially regulated *Cyp* genes in SO-HFD, HFD and Viv livers. Significantly dysregulated genes (*P* < 0.05 and *q* < 0.1) from liver RNA-seq of male C57/BL6 mice fed SO-HFD, HFD or Viv chow for 35 weeks. Given are the average FPKM values (and fold change) for three biological replicates per diet, except for HFD that had one outlier removed. Data are divided into three tabs for each comparison: “HFD v Viv,” “SO-HFD v Viv,” “SO-HFD v HFD”.
(XLSX)

S4 Dataset Significantly altered metabolites in livers of SO-HFD, HFD and Viv fed mice at 16 and 35 weeks. Metabolomic profiles of mouse liver tissue collected from C57/BL6 male mice maintained on SO-HFD, HFD and Viv chow for 16 and 35 weeks. The dataset contains 398 significantly altered (*P <* 0.1 and *q* < 0.05) biochemicals of known identity from Metabolon Inc. N = 6–8 biological replicates per condition. The various tabs contain an explanation of the file and terms (Explanation), raw data (OrigScale), imputed data (ScaledImpData), Pathway heat maps and boxplots (by pathway and by biochemical) based on both diet and time. Included are links to KEGG and Human Metabolome Database (HMDB).
(XLSX)

S1 Fig Average weekly food consumption of mice on various diets. Shown is the average amount of food consumed on a given diet measured on a per cage basis, normalized to the number of mice per cage. Food was changed and measured twice weekly; values were combined to generate the weekly average. Viv chow consumption was the highest because it has the fewest calories per gram. N = 12 mice (3–4 cages) per diet.
(TIF)

S2 Fig Additional liver sections stained with Oil Red O. Oil Red O staining for fatty liver in male mice on the various diets for 35 weeks. The HFD section at the far left is from the mouse that was an outlier in the RNAseq (Fig 6A). Scale bars are 100 microns.
(TIF)

S3 Fig Changes in liver metabolites with diet and over time. Metabolic pathway visualization (Cytoscape) of metabolomics data from livers of HFD and SO-HFD versus Viv fed male mice (n = 6–8) at 35 weeks **(A, B)** and SO-HFD versus HFD at 16 and 35 weeks (**C, D**). Circles denote significantly up-(red) and downregulated (blue) metabolites. Letters denote the metabolism nodes. **E)** Pathways showing >2-fold enrichment between the indicated treatments. Color scale: yellow (low) to red (high).
(TIF)
