# Supplementary material for: Soybean Oil Is More Obesogenic and Diabetogenic than Coconut Oil and Fructose in Mouse: Potential Role for the Liver
Source: PLoS One. 2015 Jul 22;10(7):e0132672. doi: 10.1371/journal.pone.0132672 (PMC4511588; doi:10.1371/journal.pone.0132672)
Supplement: S2 Dataset — Dysregulated genes (1.5-log(2) fold change) in SO-HFD versus HFD livers found by searching Pubmed Genes for “obesity,” “diabetes,” “inflammation” and “cancer.” Mitochondrial genes are from Mitocarta. (DOCX) [file pone.0132672.s002.docx]

**S2 Table.** Disease-related genes dysregulated (> 1.5 log fold) in RNA-seq of SO-HFD versus HFD livers.

| **Obesity** | **Diabetes** | **Inflammation** | **Mitochondrial** | **Cancer** |
| --- | --- | --- | --- | --- |
| *Adrb2* | *Adrb2* | *Abcd2* | *Abcd2* | *Adrb2* |
| *Cd36* | *Cd36* | *Adrb2* | *Acot2* | *Anxa8* |
| *Cidea* | *Cd4* | *Ccl22* | *Acyp2* | *Cd4* |
| *Ctgf* | *Cdkn1a* | *Cd36* | *Anxa5* | *Cdkn1a* |
| *Itgax* | *Ctgf* | *Cd4* | *Cd36* | *Cish* |
| *Lcn2* | *H19* | *Cd63* | *Cidea* | *Ctgf* |
| *Mt1* | *Igfbp1* | *Cdkn1a* | *Cidec* | *Dkk4* |
| *Mt2* | *Lrp2* | *Ctgf* | *Cyp2c38* | *Egfr* |
| *Retnlg* | *Mt1* | *Fhl2* | *Cyp2c39* | *Gpnmb* |
| *Socs3* | *Nt5e* | *Hmmr* | *Dact2* | *H19* |
| *Thbs1* | *Pdk4* | *Itgax* | *Grpel2* | *Hmmr* |
| *Tnc* | *Socs3* | *Lcn2* | *Hsd3b4* | *Igfbp1* |
| *Zfp423* | *Tff3* | *Lgals1* | *Hsd3b5* | *Itgax* |
|  | *Thbs1* | *Mmp12* | *Hsd3b6* | *Lcn2* |
|  |  | *Mmp13* | *Hsph1* | *Lgals1* |
|  |  | *Mt1* | *Hyou1* | *Lrp2* |
|  |  | *Mt2* | *Idh2* | *Mmp12* |
|  |  | *Npnt* | *Keg1* | *Mmp13* |
|  |  | *Nt5e* | *Lars2* | *Mt1* |
|  |  | *Plat* | *Maoa* | *Nt5e* |
|  |  | *Socs2* | *Mthfd1l* | *Onecut1* |
|  |  | *Socs3* | *Pacrg* | *Scara5* |
|  |  | *Thbs1* | *Pdk4* | *Slc43a1* |
|  |  | *Tnc* | *Serpina3c* | *Socs3* |
|  |  | *Ubd* | *Slc25a27* | *Tff3* |
|  |  | *Vldlr* | *Slc25a30* | *Thbs1* |
|  |  | *Vnn1* | *Tead1* | *Tmeff2* |
|  |  |  |  | *Tnc* |
|  |  |  |  | *Vnn1* |
|  |  |  |  | *Wif1* |

Pubmed and Mitocarta databases were searched for identifying genes related to various diseases.

**Definitions:**

**HFD:** High fat diet with 40 kcal% total fat: 36 kcal% from coconut oil and 4 kcal% from soybean oil. **SO-HFD:** Soybean-oil enriched HFD: 21 kcal% fat calories from coconut oil and 19 kcal% from soybean oil.

**Reference:**

**Soybean oil is more obesogenic and diabetogenic than coconut oil and fructose in mouse: potential role for the liver** (PLoS One,2015)

Poonamjot Deol, Jane R. Evans, Joseph Dhahbi, Karthikeyani Chellappa, Diana S Han, Stephen Spindler, Frances M. Sladek
