# Supplementary figures and images for: Soybean Oil Is More Obesogenic and Diabetogenic than Coconut Oil and Fructose in Mouse: Potential Role for the Liver
Source: PLoS One. 2015 Jul 22;10(7):e0132672. doi: 10.1371/journal.pone.0132672 (PMC4511588; doi:10.1371/journal.pone.0132672)

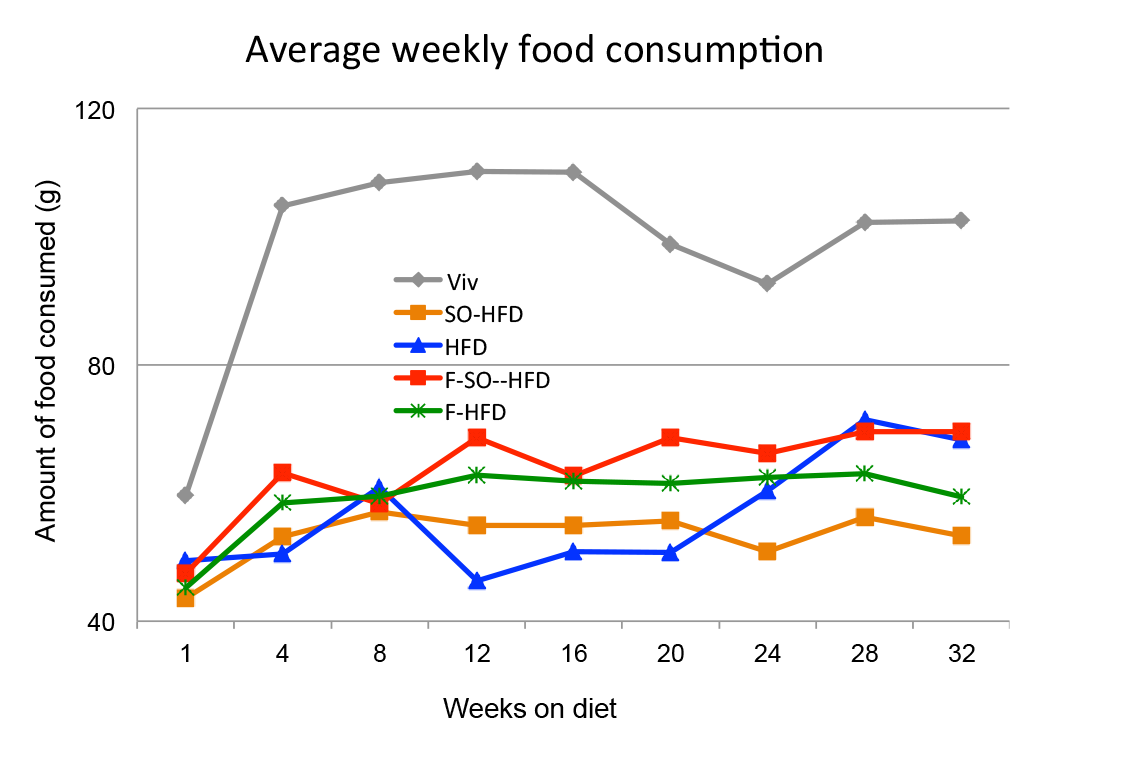

Supplement: S1 Fig — Shown is the average amount of food consumed on a given diet measured on a per cage basis, normalized to the number of mice per cage. Food was changed and measured twice weekly; values were combined to generate the weekly average. Viv chow consumption was the highest because it has the fewest calories per gram. N = 12 mice (3–4 cages) per diet. (TIF) [file pone.0132672.s005.tif]

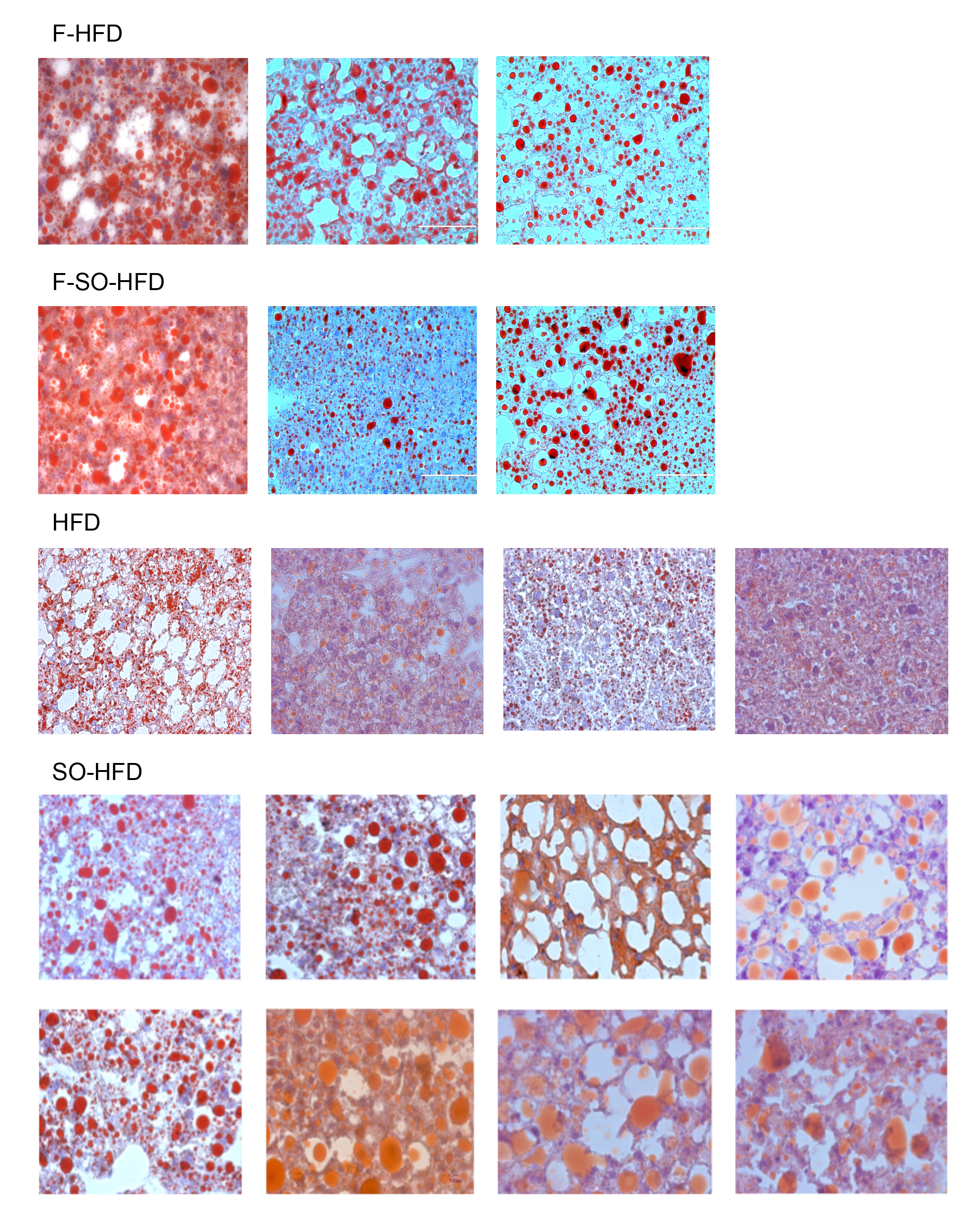

Supplement: S2 Fig — Oil Red O staining for fatty liver in male mice on the various diets for 35 weeks. The HFD section at the far left is from the mouse that was an outlier in the RNAseq (Fig 6A). Scale bars are 100 microns. (TIF) [file pone.0132672.s006.tif]

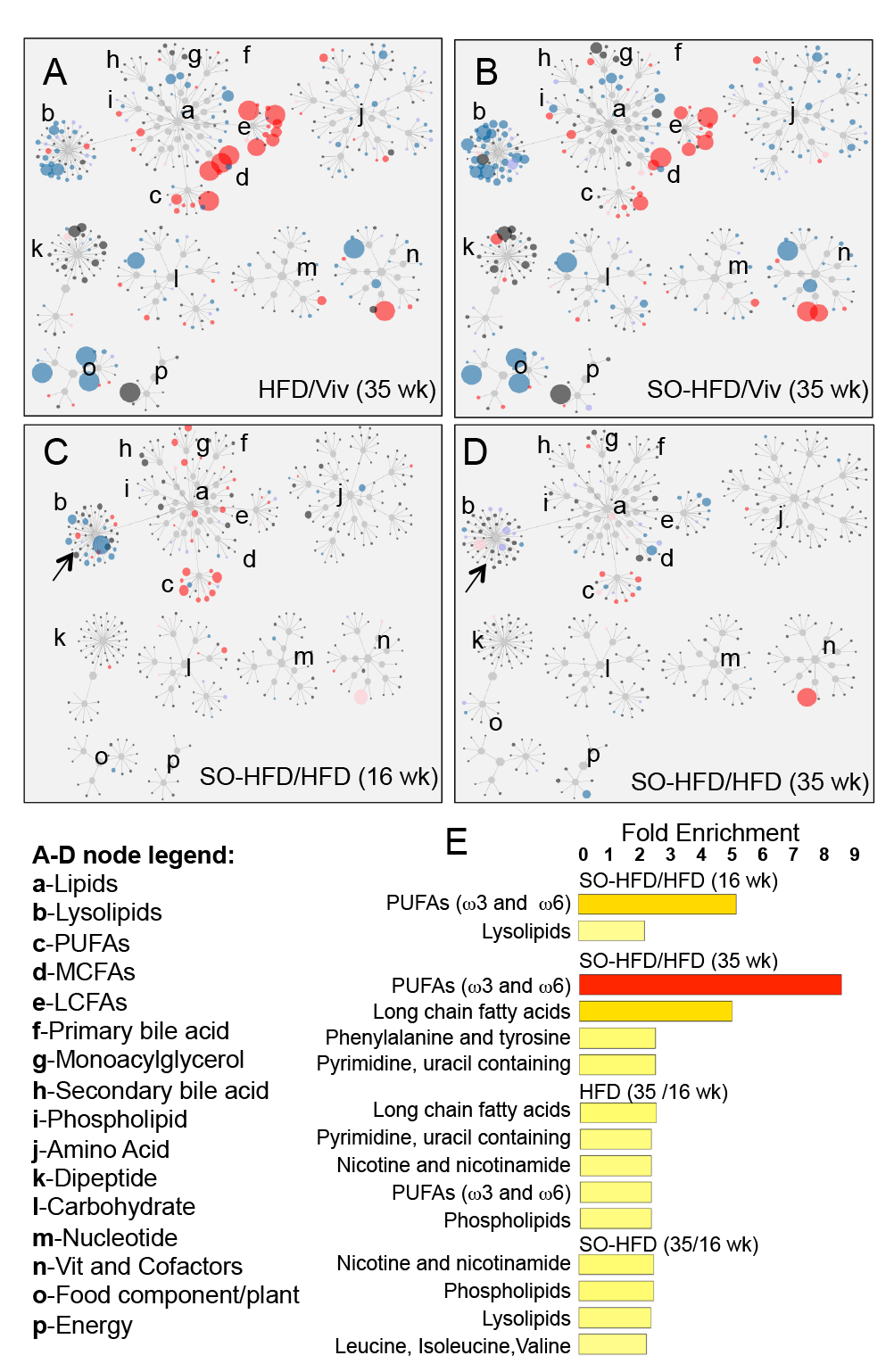

Supplement: S3 Fig — Metabolic pathway visualization (Cytoscape) of metabolomics data from livers of HFD and SO-HFD versus Viv fed male mice (n = 6–8) at 35 weeks (A, B) and SO-HFD versus HFD at 16 and 35 weeks (C, D). Circles denote significantly up-(red) and downregulated (blue) metabolites. Letters denote the metabolism nodes. E) Pathways showing >2-fold enrichment between the indicated treatments. Color scale: yellow (low) to red (high). (TIF) [file pone.0132672.s007.tif]
